# SMYD5 catalyzes histone H3 lysine 36 trimethylation at promoters

**DOI:** 10.1038/s41467-022-30940-1

**Published:** 2022-06-09

**Authors:** Yanjun Zhang, Yuan Fang, Yin Tang, Shixun Han, Junqi Jia, Xinyi Wan, Jiaqi Chen, Ying Yuan, Bin Zhao, Dong Fang

**Affiliations:** 1 Zhejiang Provincial Key Laboratory for Cancer Molecular Cell Biology, Life Sciences Institute, Zhejiang University, Hangzhou, Zhejiang 310058 China; 2 Department of Medical Oncology, Key Laboratory of Cancer Prevention and Intervention, Ministry of Education, The Second Affiliated Hospital, Zhejiang University School of Medicine, Hangzhou, Zhejiang China

**Keywords:** Histone post-translational modifications, Embryonic stem cells, Epigenetics

## Abstract

Histone marks, carriers of epigenetic information, regulate gene expression. In mammalian cells, H3K36me3 is mainly catalyzed by SETD2 at gene body regions. Here, we find that in addition to gene body regions, H3K36me3 is enriched at promoters in primary cells. Through screening, we identify SMYD5, which is recruited to chromatin by RNA polymerase II, as a methyltransferase catalyzing H3K36me3 at promoters. The enzymatic activity of SMYD5 is dependent on its C-terminal glutamic acid-rich domain. Overexpression of full-length *Smyd5*, but not the C-terminal domain-truncated *Smyd5*, restores H3K36me3 at promoters in *Smyd5* knockout cells. Furthermore, elevated *Smyd5* expression contributes to tumorigenesis in liver hepatocellular carcinoma. Together, our findings identify SMYD5 as the H3K36me3 methyltransferase at promoters that regulates gene expression, providing insights into the localization and function of H3K36me3.

## Abstract (web summary)

SETD2 methylates histone H3K36me3 in gene bodies in mammalian cells. Here the authors show H3K36me3 is also enriched at the promoter regions, and that this methylation is carried out by SMYD5, which is recruited by RNA polymerase II. They furthermore show SMYD5 is elevated in liver cancer and is correlated with changes in gene expression.

## Introduction

Nucleosomes, the basic repeating units of chromatin, consist of one core histone H3–H4 tetramer and two core histone H2A–H2B dimers, wrapping around with 146–147 base pairs of DNA^1,2^. Four core histones are extensively modified posttranslationally at their N- and C-terminal tails, including methylation, acetylation, phosphorylation, and ubiquitylation. Histone tails are heavily enriched with positively charged amino acids to interact with negatively charged DNA, and their modification can alter chromatin structure to modulate gene expression^3–5^.

H3K36 trimethylation (H3K36me3), which is deposited onto chromatin following RNA polymerase II (Pol II) elongation, usually marks active gene body regions with a gradual increase from 5’ ends toward 3’ ends of genes^6–9^. Beyond the findings that H3K36me3 is associated with actively transcribed genes^7,9^, emerging evidence indicates that H3K36me3 participates in the regulation of several cellular activities. Before DNA damage occurs, H3K36me3 could pre-recruit the DNA repair machinery to set the actively transcribed chromatin into a ‘ready’ state to quickly initiate the repair response upon DNA lesion^10^. In yeast, H3K36me3, once deposited at the active transcriptional gene bodies, recruits histone deacetylation complex to reset the activation of chromatin, leading to the prevention of cryptic transcription^11,12^. In mammalian cells, this prevention of cryptic transcription is not only mediated by the interplay between H3K36me3-mediated histone deacetylation and associated H3K4/H3K9 methylations^13,14^, but also facilitated by H3K36me3-guided DNA methylations^15–17^. Besides the regulation of mRNA levels, H3K36me3 can be recognized by METTL14 to enable the co-transcriptionally deposition of RNA m^6^A modification^18^. ZMYND11 binds H3K36me3 as an H3.3 specific reader to regulate RNA splicing and intron retention^19,20^. In addition, other regulatory functions are proposed for H3K36me3, including three-dimensional chromosome organization^21^, X-chromosome dosage compensation^22^, and pre-mRNA splicing^23^. Because of the diverse roles of H3K36me3 in cellular processes, H3K36me3 misregulations are identified in many human diseases, particularly renal cell carcinoma^24^. The H3K36me3 methyltransferase was originally identified in yeast as Set2^25,26^, and then recognized in mammalian cells as the homology SETD2^27,28^. Furthermore, meiosis-specific histone methyltransferase PRDM9 can catalyze both H3K4me3 and H3K36me3 in testis^29^.

CUT&Tag was developed to efficiently profile chromatin binding elements in cells^30^. The target protein of interest is recognized by its specific antibody which is subsequently bound with a Protein A linked Tn5 transposome. The target protein-bound DNA is cut and tagged by Tn5 transposomes to incorporate specific DNA sequences on both sides, which are then used to amplify target DNA for high-throughput sequencing. With this approach, cells are not crosslinked or sonicated so that the chromatin is maintained at a native state.

Here, we profile the distributions of H3K36me3 in primary cells and find that H3K36me3 is also enriched at promoters beyond gene body regions. Through a targeted gene screening, we find that SMYD5 catalyzes the methylation of H3K36me3 in vivo and in vitro. SMYD5 is recruited to chromatin by Pol II, resulting in the enrichment of H3K36me3 at promoters. The C-terminal domain of SMYD5 is important for its binding with histone H3 and methylation of H3K36me3. Depletion of the C-terminal domain reduces the reestablishment of H3K36me3 at promoters when *Smyd5* is overexpressed in *Smyd5* knockout (KO) cells. Moreover, elevated *Smyd5* expression contributes to the liver hepatocellular carcinoma tumorigenesis. Together, our observations uncover SMYD5 as a histone methyltransferase for H3K36me3 at promoters to control gene expression. These data expand our understanding of how H3K36me3 is catalyzed and regulated in cells.

## Results

### H3K36me3 is enriched at promoters

**Fig. 1:**
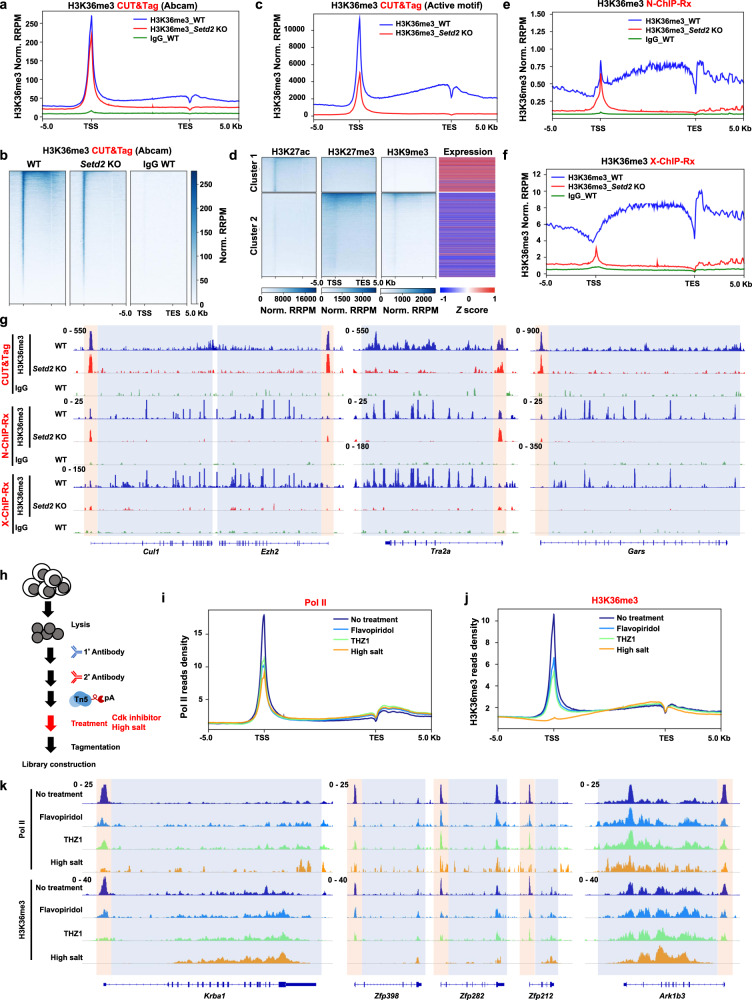
H3K36me3 is enriched at the promoters. **a** Normalized read distribution profiles of H3K36me3 CUT&Tag spanning 5 Kb of gene bodies in WT and *Setd2* KO mESCs. IgG was used as the negative control. TSS, transcription start site. TES, transcription end site. Norm. RRPM normalized reference-adjusted reads per million. **b** Heatmaps of H3K36me3 levels detected by CUT&Tag around gene body regions in WT and *Setd2* KO mESCs. 5 Kb windows spanning the TSS to TES of all genes were plotted. Genes were arranged by their enrichments of H3K36me3 in WT cells. Norm. RRPM, normalized reference-adjusted reads per million. **c** Normalized read distribution profiles of H3K36me3 CUT&Tag spanning 5 Kb of gene bodies in WT and *Setd2* KO mESCs. The anti-H3K36me3 antibody used was from Active Motif. TSS, transcription start site. TES, transcription end site. Norm. RRPM normalized reference-adjusted reads per million. **d** Heatmaps of H3K27ac, H3K27me3 and H3K9me3 CUT&Tag clustering in WT mESCs. Gene expression levels are plotted with column scaled signal scores. H3K27ac and H3K27me3 data are from GSE169049. **e** Normalized read distribution profiles of H3K36me3 N-ChIP-Rx spanning 5 Kb of gene bodies in WT and *Setd2* KO mESCs. IgG was used as the negative control. **f** As in (**e**), except H3K36me3 X-ChIP-Rx was performed. **g** IGV tracks showing the enrichment of H3K36me3 by different methods in WT and *Setd2* KO mESCs. Three different chromatin loci are shown. Red boxes: promoter regions. Blue boxes: gene body regions. **h** Schema showing the timing of adding CDK inhibitors (1 μM Flavopiridol, 1 μM THZ1) or high salt (300 mM NaCl) before tagmentation. **i** Normalized read distribution profiles of Pol II CUT&Tag spanning 5 Kb of gene bodies in WT mESCs. The average read density at all genes determined by NCBI RefSeq was plotted. Nuclei were treated with 1 μM Flavopiridol, 1 μM THZ1 or high salt (300 mM NaCl) for 30 min before tagmentation. **j** As in (**i**), except H3K36me3 CUT&Tag was performed. **k** IGV tracks showing the enrichments of Pol II and H3K36me3 by different treatments of CDK inhibitors or a high concentration of salt in WT mESCs. Three different chromatin loci were shown. Red boxes: promoter regions. Blue boxes: gene body regions.

To analyze the distribution of H3K36me3 in mouse embryonic stem cells (mESCs), we used the CRISPR/Cas9 system to knock out *Setd2* with two independent sgRNAs. Two clones were established from independent sgRNAs (Supplementary Fig. 1a). As expected, H3K36me3 was largely abolished in *Setd2* KO mESCs, as determined by Western blotting (Supplementary Fig. 1b). The total levels of other tested histone modifications were not obviously altered. We conducted H3K36me3 CUT&Tag to profile the distributions of H3K36me3 in wild-type (WT) and *Setd2* KO cells. CUT&Tag with IgG was performed in WT cells as the negative control for enrichment of H3K36me3. Two independent replicates of WT and *Setd2* KO #1 cell clones were sequenced and exhibited good correlations (Supplementary Table 1). We merged the two replicates and used *E.coli* DNA, which was carried over by the Tn5 transposomes in CUT&Tag, as the spike-in control to normalize the sequencing results among different samples for downstream analysis. Then, we analyzed enrichment of H3K36me3 in the region encompassing 5 Kb upstream and downstream of gene body regions. As previously reported^24^, the H3K36me3 level showed a sequential increase from the transcription start site (TSS) to the transcription end site (TES) in genes that were identified by NCBI RefSeq in WT cells (Fig. 1a, b). This H3K36me3 enrichment at gene body regions was largely abolished when *Setd2* was knocked out. Surprisingly, we detected strong enrichment of H3K36me3 at promoters in WT and *Setd2* KO cells, that was not observed before. Deficiency of *Setd2* led to a slight decrease but not a total removal of H3K36me3 at promoters. Although the antibody we used was previously reported to specifically bind to H3K36me3, we used the other previously validated anti-H3K36me3 antibody (from Active Motif) and performed CUT&Tag to exclude the possibility of nonspecific binding of the antibodies used^31–33^. Two replicates with high correlations were conducted and merged for analysis (Supplementary Table 1). Enrichment of H3K36me3 at gene body regions was abolished in *Setd2* KO cells when compared to WT cells. Similarly, we detected reduced enrichment but not a complete absence of H3K36me3 at promoters in *Setd2* KO cells (Fig. 1c and Supplementary Fig. 1c, d). It’s unlikely that nonspecificity of the antibodies led to the detected signal of H3K36me3 at promoters. In addition, we normalized the CUT&Tag signal without spike-in *E.coli* DNA to avoid over-normalization. We also detected strong enrichment of H3K36me3 at promoters in both WT and *Setd2* KO mESCs using two different antibodies (Supplementary Fig. 1e, f). Moreover, in previous studies, low intensities of DNA signals at promoters were detected by sequential RSC (remodeling the structure of chromatin) CUT&RUN and H3K36me3 ChIP-seq in yeast, further confirming the existence of H3K36me3 at promoters^34^.

The Tn5 transposome, which is also used in ATAC-seq (assay for transposase-accessible chromatin using sequencing) to profile the accessibility of chromatin, can attack open chromatin regions, leading to nonspecific signals at promoters in an open state. To investigate this possibility, we analyzed the H3K27ac and H3K27me3 CUT&Tag results in WT mESCs from the GEO dataset GSE169049. Because H3K27me3 was largely enriched on chromatin, and the antibody against H3K27me3 was unlikely to cause open chromatin bias, we performed H3K9me3 CUT&Tag as a complementary analysis of repressive histone mark. Moreover, we included the RNA-seq results to compare gene expression levels. H3K27me3 and H3K9me3 were detected at the promoters of weakly expressed genes, and H3K27ac was found at the promoters of genes with high expression levels (Fig. 1d). More importantly, H3K27me3 and H3K9me3 CUT&Tag signals were not enriched at the active gene promoters, which were in an open state for transposome access, suggesting that the Tn5 transposome was not randomly targeted to open chromatin regions in the CUT&Tag experiments.

Furthermore, we sought to profile the enrichment of H3K36me3 on chromatin by a classical ChIP-seq analysis. We performed native H3K36me3 ChIP-seq with a reference exogenous genome (N-ChIP-Rx) in WT and *Setd2* KO cells. Chromatin was digested by MNase without crosslinking or sonication. Chromatin from *Drosophila* S2 cells was used as the exogenous spike-in genome to normalize the sequencing signals. The two biological replicates for each sequencing run were highly correlated and were merged for further analysis (Supplementary Table 1). As expected, H3K36me3 at gene body regions was largely abolished in *Setd2* KO cells (Fig. 1e and Supplementary Fig. 1g). Interestingly, we detected enrichment of H3K36me3 at promoters in WT and *Setd2* KO cells, similar to the results of H3K36me3 CUT&Tag. There was a slight decrease of H3K36me3 at promoters in *Setd2* KO cells compared with WT cells. In addition, we analyzed the H3K36me3 ENCODE data in mESCs (ENCSR000CGR) and found weak enrichment at promoters, further supporting the idea that H3K36me3 is enriched at promoters (Supplementary Fig. 1h, i). Because these approaches, including the H3K36me3 ENCODE project, were based on noncrosslinked cells, we speculated that enrichment of H3K36me3 at promoters is detected only in native cells. To test this idea, we conducted crosslinking ChIP-seq with a reference exogenous genome (X-ChIP-Rx) in WT and *Setd2* KO cells. Chromatin was crosslinked before MNase digestion and sheared by sonication. Chromatin from *Drosophila* S2 cells was spiked in for sequencing data normalization. Two biological replicates, which were highly correlated (Supplementary Table 1), were merged for further analysis. Consistent with previous observations, H3K36me3 was enriched in gene body regions, with a gradual increase with proximity to the 3’ end (Fig. 1f and Supplementary Fig. 1j). In particular, we did not detect enrichment of H3K36me3 at promoters in WT cells. In addition, only a very weak H3K36me3 signal intensity was detected at promoters in *Setd2* KO cells. Furthermore, visualization with IGV (Integrative Genomics Viewer) revealed that H3K36me3 was enriched at promoters in the CUT&Tag and N-ChIP-Rx data but not in the X-ChIP-Rx data (Fig. 1g). These data indicate that there is enrichment of H3K36me3 at promoters that is not dependent on SETD2 and that this enriched H3K36me3 is unstable to be quickly released from the chromatin when cells are crosslinked and sonicated. In support of this idea, the relative histone occupancy at TSSs compared to the surrounding regions was less in X-ChIP-Rx data than in N-ChIP-Rx data (Supplementary Fig. 1k, l).

The enriched H3K36me3 was located at promoters which were also highly occupied by Pol II. In addition, a high concentration of salt (300 mM NaCl) reduced the nonspecific tagmentation by the Tn5 transposome at active histone marks^35,36^. To elucidate whether Pol II can affect the enrichment of H3K36me3 at promoters, we used CDK inhibitors or a high concentration of salt (300 mM NaCl) to remove Pol II from chromatin after antibody and Tn5 transposome binding and then proceeded to perform tagmentation (Fig. 1h)^30^. Cells were treated with two CDK inhibitors—Flavopiridol, which is a CDK9 inhibitor, and THZ1, which is a CDK7 inhibitor—at a concentration of 1 μM for 30 min to remove Pol II from promoters. Two biological replicates with high correlation between each sequencing run were performed and merged for further analysis (Supplementary Table 1). We detected a decrease in Pol II occupancy at promoters when samples were treated with CDK inhibitors or a high concentration of salt (Fig. 1i and Supplementary Fig. 1m). Interestingly, we observed that H3K36me3 enrichment was reduced at promoters when CDK inhibitors or a high concentration of salt were used (Fig. 1j and Supplementary Fig. 1n). These observations were also revealed by visualization of three representative genomic regions with IGV (Fig. 1k). Notably, a greater reduction in H3K36me3 at promoters was detected when samples were treated with a high concentration of salt. A high concentration of salt can induce the release of many proteins in addition to Pol II from chromatin, and it is possible that these released proteins, including Pol II, were also responsible for enrichment of H3K36me3 at promoters. Together, these data suggest that H3K36me3 is enriched at promoters in native cells and that this enrichment is regulated by Pol II.

### SMYD5 catalyzes H3K36me3

**Fig. 2:**
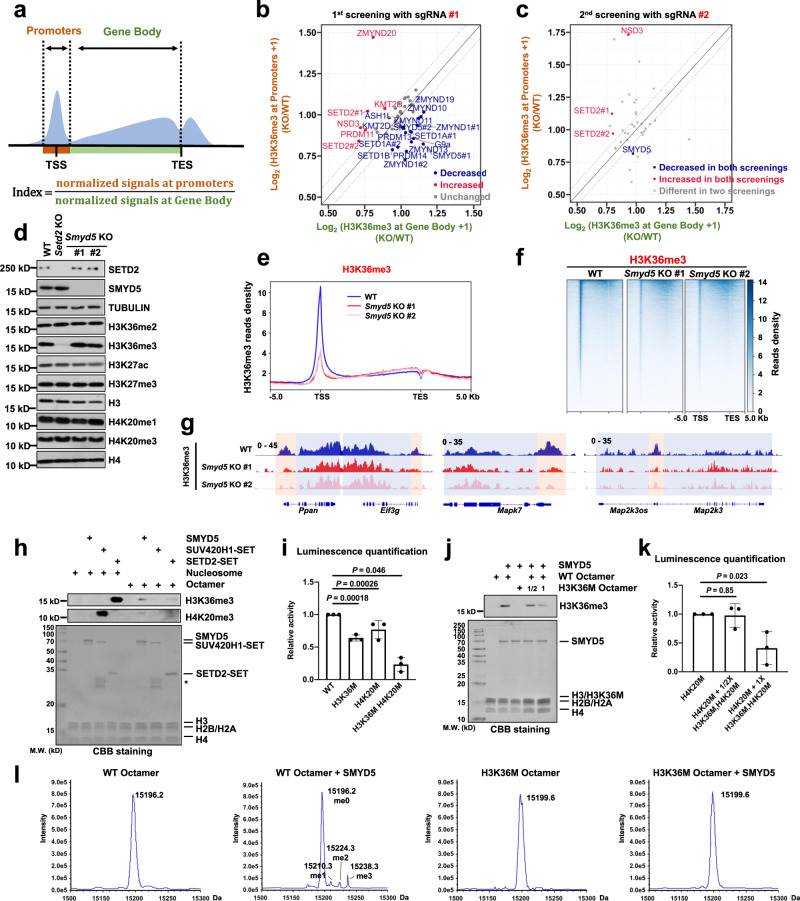
SMYD5 methylates H3 to H3K36me3. **a** Schema showing the calculation of the H3K36me3 index. **b** Dot plot showing the first screening results. Genes, KO of which changed H3K36me3 indexes less than 15% (gray), decreased or increased the H3K36me3 indexes in blue and red, respectively. **c** Dot plot showing the second screening results. color scheme as in (**b**). **d** Protein levels in WT and *Smyd5* KO mESCs. *Setd2* KO #1 mESCs were used as a positive control for H3K36me3. Cell extracts were analyzed by Western blotting using the specified antibodies. Two independent experiments were performed. Source data are provided as a Source Data file. **e** The normalized read distribution profiles of H3K36me3 CUT&Tag spanning 5 Kb of gene bodies in WT and *Smyd5* KO mESCs. TSS, transcription start site. TES, transcription end site. **f** Heatmaps of H3K36me3 levels detected by CUT&Tag around gene body regions in WT and *Smyd5* KO mESCs. 5 Kb windows spanning the TSS to TES of all genes determined by NCBI RefSeq were plotted. **g** IGV tracks presenting the enrichment of H3K36me3 by H3K36me3 CUT&Tag in WT and *Smyd5* KO mESCs. Red boxes: promoter regions. Blue boxes: gene body regions. **h** HMT assays of full length SMYD5, SET domain of SUV420H1, and SET domain of SETD2 proteins against nucleosome or octamer substrates. Upper panel: Western blotting. Lower panel: the input by Coomassie Brilliant Blue (CBB) staining. Each assay was repeated at least three times with similar results. Star indicated the non-specific proteins. **i** End-point HMT assays of SMYD5 against an equal amount of WT, H3K36M, H4K20M, and H3K36M, H4K20M mutant octamers. *N* = 3 independent experiments. Data are mean ± SD. *P* values were calculated by one-way ANOVA. **j** HMT assays of full length SMYD5 against different amounts of WT and H3K36M octamer substrates. Upper panel: H3K36me3 western blot. Lower panel, input by Coomassie Brilliant Blue (CBB) staining. Each assay was repeated at least three times with similar results. Source data are provided as a Source Data file. **k** End-point HMT assays of SMYD5 against the different amounts of H4K20M and H3K36M/H4K20M octamers. *N* = 3 independent experiments. Data are mean ± SD. *P* values were calculated by one-way ANOVA. Source data are provided as a Source Data file. **l** ESI-TOF mass spectrometry analysis of Histone H3.

We then sought to determine which methyltransferase is responsible for the enrichment of H3K36me3 at promoters. Because the enriched H3K36me3 at promoters was location-specific and may only account for a small amount of total H3K36me3, we established cell lines with knockout of potential methyltransferases and then performed H3K36me3 CUT&Tag to directly analyze the changes in H3K36me3 at promoters. For this screening, we selected known H3K36 methyltransferases; major histone methyltransferases that contain SET domains and are responsible for depositing histone marks at promoters; PRDM family proteins, which contain SET domains; SMYD family proteins, which are reported to be responsible for H3K4me3 deposition at promoters; and ZMYND family proteins, which are potential H3K36 methylation readers. Under these criteria, 37 genes were selected. One sgRNA was used to knock out the target gene in the first screen. Single clones were generated, and gene KO was confirmed by Sanger sequencing. Because H3K36me3 was located at promoters and in gene body regions, we used an H3K36me3 index to compare enrichments between these two regions and to normalize signals in different batches of experiments. Thirteen batches of experiments were conducted. Signals in gene KO cells were normalized to those in WT cells in the same batch of experiments (Fig. 2a). For normalization of the H3K36me3 index in KO cells to that in WT cells, values less than 1 indicated lower enrichment at promoters, whereas values larger than 1 indicated higher enrichment at promoters. Using a change of 15% in the index as the criterion, we found that cell with individual knockout of 5 genes, including *Setd2* with two repeats, showed a relatively increased in H3K36me3 at promoters and that cells with individual knockout of 14 genes, including two repeats of *Smyd5* and *Zmynd1*, exhibited decreased H3K36me3 at promoters (Fig. 2b and Supplementary Table 2). We then selected these genes and genes that led to a change close to the 15% cutoff for further screening. A total of 32 genes were selected for the second screen. To increase the reproducibility of the screening results, we used the other independent sgRNA for each gene and generated single clones for analysis by H3K36me3 CUT&Tag. From these two screens, we found that compared with the parental cells, only *Nsd3* KO cells showed a consistently increase in H3K36me3 at promoters and *Smyd5* KO cells exhibited decreased H3K36me3 at promoters (Fig. 2c and Supplementary Table 3). These data indicate that *Smyd5* is one of the enzymes responsible for enrichment of H3K36me3 at promoters.

Two *Smyd5* KO cell lines were generated from two independent sgRNAs bearing 5 and 7 base pair deletions, respectively (Supplementary Fig. 2a). While *Setd2* KO dramatically decreased H3K36me3 in mESCs, *Smyd5* KO didn’t change the total level of H3K36me3 to a detectable level, as determined by Western blotting (Fig. 2d). The total levels of other analyzed histone marks, including H4K20me3, which was previously reported to be methylated by SMYD5^37^, were not changed to detectable levels. In addition, *Smyd5* KO didn’t affect the cell proliferation or alkaline phosphatases activity in mESCs (Supplementary Fig. 2b, c). In addition, SMYD5 was localized in both the nucleus and cytoplasm. When knockdown *Smyd5* in Hela cells, SMYD5 signal decreased largely in cytoplasm, though SMYD5 were not co-stain with Mitochondria (Supplementary Fig. 2d, e). The localization of SMYD5 is further confirmed by Western blotting (Supplementary Fig. 2f). There are possible nonchromatin substrates of SMYD5, as SMYD5 is distributed in the cytoplasm. We further analyzed the enrichment of H3K36me3 in genomic regions spanning 5 Kb upstream and downstream of the gene body region in all genes, which were identified by NCBI RefSeq, in WT and *Smyd5* KO mESCs. The two independent replicates of each KO cell line were highly correlated and were merged for further analysis (Supplementary Table 1). Consistent with the screening results, H3K36me3 was decreased at promoters in both *Smyd5* KO clones (Fig. 2e, f). The decrease in H3K36me3 at promoters was revealed by visualization of three representative chromatin loci with IGV (Fig. 2g). Notably, enrichment of H3K36me3 at promoters was not fully abolished when *Smyd5* was knocked out. It reamains possible that there are additional enzymes and regulators responsible for this specific H3K36me3 enrichment.

To further confirm whether SMYD5 can methylate H3K36, we tested the enzymatic activities of SMYD5 in vitro by a histone methyltransferase (HMT) assay. Since the specific H3K36me3 was detected at the promoters and found to undergo highly dynamic changes, it is possible that H3 was not assembled into nucleosomes when it was methylated by SMYD5. We used both reconstituted core nucleosomes and core octamers as the substrate. The SET domains of SETD2 and SUV420H1 were used as positive controls for H3K36me3 and H4K20me3, respectively. SMYD5 catalyzed H3K36me3 using core octamers but not core nucleosomes as the substrate (Fig. 2h and Supplementary Fig. 2g). As previously reported, SMYD5 catalyzed H4K20me3 using core octamers as the substrate^37^, and SETD2 and SUV420H1 preferentially catalyzed histone methylation using core nucleosomes as the substrate^38,39^. Furthermore, the total methyltransferase activity of SMYD5 decreased to approximately 60%, 80%, and 25% when H3K36M, H4K20M and H3K36M, H4K20M mutant octamers were used as substrates, respectively, further confirming that SMYD5 exhibited enzymatic activity toward the H3K36 and H4K20 sites (Fig. 2i). We and others have previously found that the H3K36M mutant protein can inhibit the enzymatic activity of H3K36 methyltransferases^31,32^. Therefore, we analyzed whether the enzymatic activity of SMYD5 is suppressed by mixing WT octamers with different amounts of H3K36M mutant octamers. When H3K36M mutant octamers were added at half the amount of WT octamers, the enzymatic activity of SMYD5 was slightly inhibited (Fig. 2j). Methyltransferase activity was largely suppressed when the amount of H3K36M mutant octamers increased to the same level as WT octamers. To further quantify this inhibitory effect, we used H4K20M octamers, which would not be modified to H4K20me3, as the substrate and then added different amounts of H3K36M, H4K20M double mutant octamers. Consistent with the above findings, the double mutant octamers suppressed the methyltransferase activity of SMYD5 when the same amount of H4K20M and H3K36M, H4K20M octamers were mixed and used as the substrate (Fig. 2k). To further confirm the specific site of histone H3 methylation in the presence of SMYD5, we determined the molecular weight of H3 after the in vitro HMT assay by mass spectrometry. SMYD5 tri-methylated WT but not K36M mutant H3 when octamers were used as the substrates (Fig. 2l). Together, these results suggest that SMYD5 can methylate H3K36me3 using core octamers as the substrate and is one of the methyltransferases catalyzing H3K36me3 at promoters.

### SMYD5 is enriched at the promoters to regulate H3K36me3 and gene expression

**Fig. 3:**
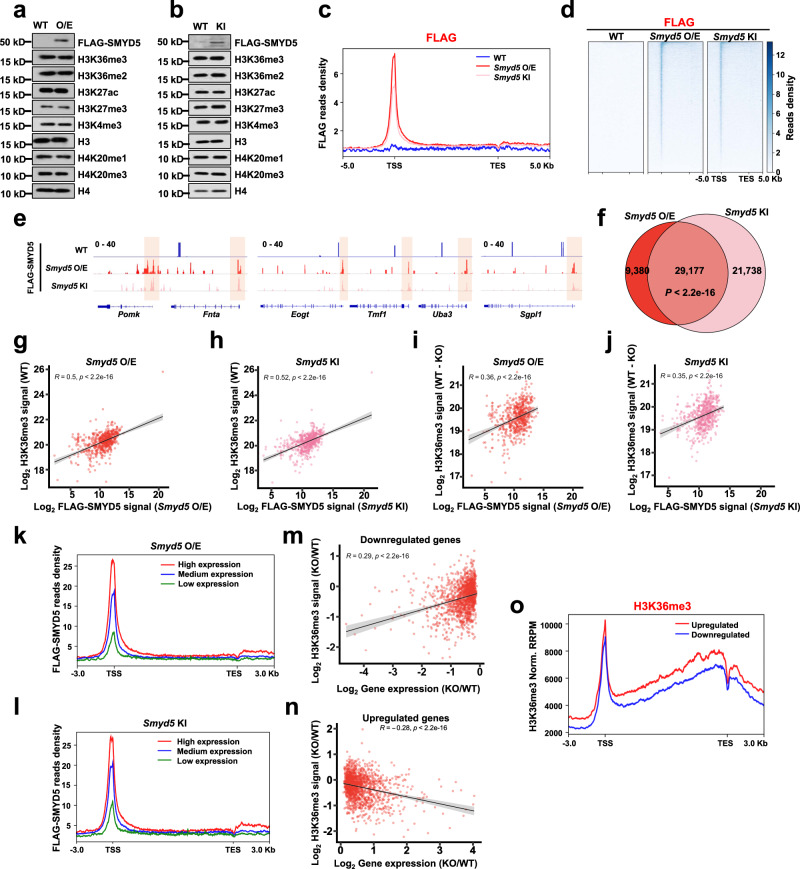
SMYD5 localizes at promoters to methylate H3K36me3 and regulate gene expression. **a** Overexpression of *Smyd5* does not alter the levels of tested histone marks. O/E, *FLAG-Smyd5* overexpression. Two independent experiments were performed. **b** Knock-in of a FLAG tag at the 5’ end of *Smyd5* does not alter the levels of tested histone marks. KI, knock-in of a FLAG tag at the 5’ end of *Smyd5*. Two independent experiments were performed. **c** Normalized read distribution profiles of FLAG CUT&Tag spanning 5 Kb of gene bodies in WT, *FLAG-Smyd5* overexpression, and FLAG-tag knock-in *Smyd5* mESCs. TSS, transcription start site. TES, transcription end site. O/E, over-expression. KI, FLAG tag knock-in. **d** Heatmaps of FLAG levels detected by CUT&Tag around gene body regions. 5 Kb windows spanning the TSS to TES of all genes were plotted. **e** IGV tracks presenting the enrichments of FLAG-tagged proteins by FLAG CUT&Tag. Three different chromatin loci are shown. Red boxes indicated the promoter regions. **f** Venn diagram illustrating the overlap of FLAG-SMYD5 peaks detected in *FLAG-Smyd5* over-expression and FLAG-tag knock-in mESCs. *P* value was determined by Fisher’s exact statistical test, two-sided. **g** Correlations between the signals of H3K36me3 and FLAG-SMYD5 at promoters in *FLAG-Smyd5* overexpression mESCs. Each dot indicates a single promoter. R, correlation coefficients that were assessed by Pearson product moment correlation. *P* values were calculated by paired *t* test, two-sided. **h** Same as in (**g**), except mESCs with a FLAG tag at the 5’ end of *Smyd5* were used. **i** Correlations between the signals of FLAG-SMYD5 and alternations of H3K36me3 at the promoters in *FLAG-Smyd5* over-expression mESCs. Each dot indicates a single promoter. R, correlation coefficients that were assessed by Pearson product moment correlation. *P* values were calculated by paired *t* test, two-sided. **j** Same as in (**i**), except mESCs with a FLAG tag at the 5’ end of *Smyd5* were used. **k** Normalized read density of FLAG-SMYD5 from 3 Kb upstream of the TSS to 3 Kb downstream of the TES in grouped genes with high, medium, and low expression levels in *FLAG-Smyd5* over-expression mESCs. Genes were separated into high, medium, and low expression groups based on their expression levels in WT mESCs. High expression, expression levels at top 25%. Low expression, expression levels at bottom 25%. Medium expression, expression levels between high and low expression groups. **l** Same as in (**k**), except mESCs with a FLAG tag at the 5’ end of *Smyd5* were used. **m** The difference in normalized read densities of H3K36me3 at promoters between parental and *Smyd5* KO mESCs relative to alternations of gene expression. Genes that were downregulated in *Smyd5* KO cells (*P* < 0.05) comparing to parental cells are plotted. R, correlation coefficients that were assessed by Pearson product moment correlation. Confidence interval shows the SEM. **n** Same as in (**m**), except genes that were upregulated in *Smyd5* KO cells (*P* < 0.05) comparing to parental cells are plotted. Confidence interval shows the SEM. **o** Normalized read density of H3K36me3 from 3 Kb upstream of the TSS to 3 Kb downstream of the TES in grouped genes, which were upregulated or downregulated in *Smyd5* KO mESCs. Genes were separated into upregulated genes or downregulated genes as defined in (**m**).

We attempted three CUT&Tag analyses with the anti-SMYD5 antibody but did not detect specific signals when comparing the signals between WT and *Smyd5* KO #1 mESCs (Supplementary Fig. 3a). To specifically analyze how SMYD5 was localized on chromatin, we overexpressed FLAG-tagged *Smyd5* and knocked in a FLAG tag at the N- terminus of the endogenous *Smyd5* sequence (Supplementary Fig. 3b). In these two cell lines, the total levels of H3K36me3 and H4K20me3 were not altered to detectable levels, as determined by Western blotting (Fig. 3a, b). In addition, the levels of other tested histone marks were not changed. We then performed FLAG CUT&Tag to profile the distribution of SMYD5 on chromatin. WT mESCs were used as the negative control cells. Two independent replicates with high correlations were conducted and merged for further analysis (Supplementary Table 1). Exogenously and endogenously expressed SMYD5 were enriched at promoters (Fig. 3c, d). No specific signals were detected in WT mESCs, confirming the specificity of FLAG CUT&Tag. Individual IGV visualizations also revealed that overexpressed and N-terminal FLAG-tagged SMYD5 were enriched at promoters at three representative chromatin loci (Fig. 3e). In addition, we performed FLAG CUT&Tag with *Smyd5* overexpressed cells treated with a high salt concentration (300 mM NaCl). No specific signals were detected in gene bodies or at promoter regions after the high salt treatment (Supplementary Fig. 3c), consistent with the previous observation that high salt treatment reduced the enrichment of H3K36me3 at promoters (Fig. 1j).

To evaluate the distribution of exogenously and endogenously expressed SMYD5 on chromatin, we called FLAG peaks and obtained 38,557 and 50,915 peaks for exogenous and endogenous SMYD5, respectively. A total of 29,177 peaks overlapped significantly, suggesting that overexpressed SMYD5 and endogenous SMYD5 were localized at similar chromatin loci (Fig. 3f). We further compared the colocalization of SMYD5 and H3K36me3 at promoters. To increase the strength of the comparison, we used overlapped peaks of exogenous and endogenous SMYD5 at promoters and compared them with H3K36me3 peaks that overlapped with promoters. A large number of SMYD5 and H3K36me3 peaks overlapped at the promoters (Supplementary Fig. 3d). We download the biotin-SMYD5 ChIP-seq data in mESC from GEO dataset GSE94086. We then combined the ChIP-seq results with our CUT&Tag results to further validate the target genes of SMYD5. The peaks of SMYD5 ChIP-seq and CUT&Tag signals overlapped well and the signals of SMYD5 ChIP-seq were localized at the overlapped peaks of SMYD5 CUT&Tag, indicating SMYD5 were enriched at these peaks and were detected by different ways (Supplementary Fig. 3e, f). The colocalization of SMYD5 and H3K36me3 at promoters prompted us to evaluate their correlations at promoters more carefully. We plotted the signals of H3K36me3 and overexpressed SMYD5 at promoters that overlapped with the H3K36me3 peaks in WT mESCs. We observed a strong positive correlation (R = 0.5) between the signals of SMYD5 and H3K36me3 at promoters (Fig. 3g). Under the same conditions, we also found a high correlation (R = 0.52) between H3K36me3 and endogenous FLAG-tagged SMYD5 (Fig. 3h). Moreover, we plotted the alternations in H3K36me3 signals between WT and *Smyd5* KO #1 cells with SMYD5 signals at promoters that overlapped with the H3K36me3 peaks. Strong positive correlations were detected between the changes in the H3K36me3 signals and SMYD5 signals, suggesting that the alterations in H3K36me3 were directly associated with *Smyd5* KO (Fig. 3i, j). We also analyzed the genome-wide effects of overexpression of *Smyd5* on H3K36me3, Pol II and H4K20me3 by CUT&Tag. Two independent replicates with high correlations were conducted and merged for further analysis (Supplementary Table 1). H3K36me3 increased slightly at the promoters with overexpressed SMYD5, whereas Pol II and H4K20me3 were not changed at these promoters (Supplementary Fig. 3g–m). It is possible that other H4K20me3 methyltransferase enzymes, including SUV420H1 and SUV420H2, were also enriched at promoters to compensatorily deposit H4K20me3. Moreover, we overexpressed *Smyd5* in K562 cells and performed H3K36me3 CUT&Tag (Supplementary Fig. 3n–p). The overexpression of *Smyd5* increased the levels of H3K36me3 at TSS regions whereas the H3K36me3 was not altered in gene body regions.

H3K36me3 was previously reported to suppress spurious transcription initiation^25^. We performed GRO-seq (Global Run-On sequencing) to quantify nascent RNA in WT and *Smyd5* KO mESCs. Two replicates with yeast RNA as the spike-in control were conducted and showed high correlations in the WT cell line and two *Smyd5* KO cell lines (Supplementary Fig. 4a). The average levels of accumulated nascent RNA (both the sense and antisense strands) were not altered when *Smyd5* was knocked out, suggesting that loss of SMYD5-mediated H3K36me3 did not affect spurious transcription initiation (Supplementary Fig. 4b). We further compared the gene expression profiles in WT and *Smyd5* KO #1 cells by RNA-seq. Two independent replicates of WT and *Smyd5* KO #1 cells were sequenced and exhibited good correlations (Supplementary Fig. 4c). A total of 921 and 828 genes were up- and downregulated, respectively, in *Smyd5* KO cells compared with WT cells when absolute log_2_(*fold change*) > 0.5 and *P* < 0.05 were set as the cutoff thresholds (Supplementary Fig. 4d). We performed separate Gene Ontology (GO) analyses using genes that were identified as up- and downregulated in *Smyd5* KO cells (Supplementary Fig. 4e). The downregulated genes were enriched in the terms of cell adhesion, Ras signaling, Wnt signaling, and liver development, while the upregulated genes were mainly enriched in the terms of metabolic process and heart morphogenesis terms. To reveal how SMYD5 occupancy is correlated with gene expression, we divided the genes into the high, medium, and low expression groups and plotted the distributions of SMYD5 around these genes^40^. The detected SMYD5 signals were highly enriched in actively transcribed genes in both SMYD5-overexpressing and FLAG tag knock-in cells (Fig. 3k, l).

We found similar numbers of down- and upregulated genes in *Smyd5* KO cells. This was similar to the pattern after knockdown of the H3.3K36me3 reader ZMYND11, which led to both decreases and increases in gene expression^19,20^. We then separated the down- and upregulated genes to evaluate the correlations between gene expression and H3K36me3 at promoters in more detail. We plotted the alterations in gene expression between WT and *Smyd5* KO #1 cells and H3K36me3 at promoters that overlapped with H3K36me3 peaks in WT cells (Fig. 3m, n). We observed a strong positive correlation (R = 0.29) between the changes in downregulated genes and H3K36me3 levels and a strong negative correlation (R = −0.28) between the alterations in upregulated genes and H3K36me3 levels. Moreover, the upregulated genes were associated with high H3K36me3 levels, and the downregulated genes were associated with low H3K36me3 levels (Fig. 3o and Supplementary Fig. 4f). It is possible that SMYD5 safeguarded the highly expressed genes to be further activated and acted as an accelerator to promote the expression of weakly expressed genes. Collectively, these data indicate that SMYD5 is enriched at promoters to deposit H3K36me3 and regulate gene expression.

### Pol II interacts with SMYD5 and regulates its enzymatic activity

**Fig. 4:**
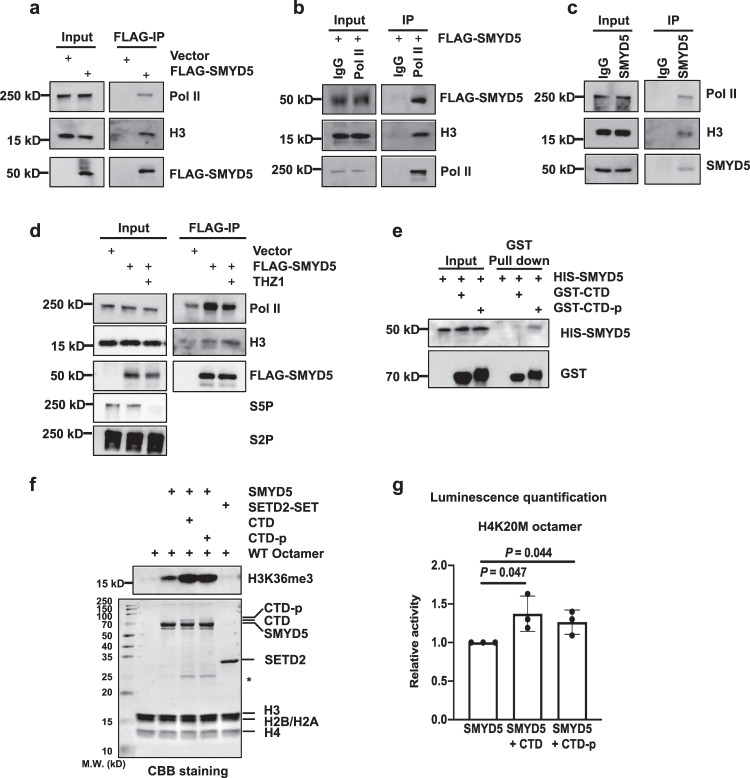
Pol II interacts with SMYD5 and regulates its enzymatic activity toward H3K36me3. **a** Over-expressed SMYD5 interacts with Pol II and H3. SMYD5 was purified by FLAG IP from HEK 293 T over-expressing FLAG-tagged SMYD5. HEK 293 T transfected with empty vectors were used as negative controls. Two independent experiments were performed. **b** Pol II bound with H3 and overexpressed SMYD5. Pol II was purified by Pol II antibody from HEK 293 T over-expressing FLAG-tagged SMYD5. IgG antibody was used as the negative control. Two independent experiments were performed. **c** Endogenous SMYD5 interacts with Pol II and H3. SMYD5 was purified by SMYD5 antibody from WT HEK 293 T. IgG antibody was used as the negative control. Two independent experiments were performed. **d** THZ1 treatment decreased the interaction between SMYD5 and Pol II. SMYD5 was purified by FLAG IP from HEK 293 T overexpressing FLAG-tagged SMYD5. THZ1 treatment was 1 μM for 30 min. Two independent experiments were performed. **e** Phosphorylation of Pol II CTD increased its interaction with SMYD5 in vitro. GST tagged Pol II CTD was purified and then phosphorylated by the CDK7-Cyclin H complex. Recombinant HIS-tagged SMYD5 was purified and incubated with phosphorylated or unphosphorylated Pol II CTD. GST Seflnose Resin beads were used to pull down Pol II CTD. CTD, Pol II CTD. CTD-p, phosphorylated Pol II CTD. *P* values were calculated by Hypergeometric distribution test, two-sided, adjusted by BH adjustment for multiple comparisons. Two independent experiments were performed. **f** HMT assays of full length SMYD5 against octamer substrates in the presence of Pol II CTD or phosphorylated Pol II CTD. Upper panel, H3K36me3. Lower panel, input by Coomassie Brilliant Blue (CBB) staining. Each assay was repeated at least three times with similar results. Star indicated the non-specific proteins. CTD, Pol II CTD. CTD-p, phosphorylated Pol II CTD. **g** End-point HMT assays of SMYD5 against an equal amount of H4K20M mutant octamers. Phosphorylated or unphosphorylated Pol II CTD was added to determine the changes of enzymatic activities of SMYD5. After the reaction, SAM was transferred to SAH which was detected by the MTase-Glo™ assay. *N* = 3 independent experiments. Data are mean ± SD. *P* values were calculated by one-way ANOVA. CTD, Pol II CTD. CTD-p, phosphorylated Pol II CTD. Source data are provided as a Source Data file.

SMYD5-mediated H3K36me3 was enriched at promoters, and this enrichment was reduced by inhibition of Pol II, indicating that SMYD5 is regulated by Pol II to methylate H3. To test this hypothesis, we overexpressed FLAG-tagged SMYD5 in HEK 293 T cells and performed immunoprecipitation (IP) using anti-FLAG beads. Pol II and H3 were copurified with FLAG-SMYD5, as detected by Western blotting (Fig. 4a). In addition, FLAG-tagged SMYD5 and H3 were copurified with Pol II when anti-Pol II antibodies were used (Fig. 4b). To further confirm these interactions, we used anti-SMYD5 antibodies and found that Pol II and H3 were copurified with endogenous SMYD5 (Fig. 4c).

We then investigated whether the interactions among SMYD5, H3, and Pol II were regulated by the phosphorylation states of Pol II. THZ1 is a CDK7 inhibitor that suppresses serine 5 phosphorylation (S5P) in the Pol II C-terminal domain (CTD). After treatment with 1 μM THZ1 for 30 min, S5P was inhibited without significant suppression of serine 2 phosphorylation (S2P) in the Pol II CTD (Fig. 4d). The interaction between SMYD5 and Pol II was obviously inhibited upon THZ1 treatment, although weak background binding of Pol II was detected. The interaction between H3 and SMYD5 was not detectably affected. To further confirm these results, we sought to conduct an in vitro pulldown assay by using purified SMYD5 proteins and Pol II CTD proteins. In addition, we purified the CDK7-Cyclin H complex and CDK9-Cyclin T1 complex for phosphorylation of the Pol II CTD. As previously reported^41,42^, both complexes induced S5P of the Pol II CTD, and the CDK7-Cyclin H complex showed lower enzymatic activity for S2P in the Pol II CTD in vitro (Supplementary Fig. 5a, b). We used the CDK7-Cyclin H complex to phosphorylate the Pol II CTD, and analyzed the interactions between SMYD5 and the Pol II CTD in vitro. Consistent with the in vivo IP results, phosphorylation increased the interaction between the Pol II CTD and SMYD5 (Fig. 4e).

Since the Pol II CTD can bind to SMYD5, we next sought to determine whether this interaction affects the enzymatic activity of SMYD5. The H3K36me3 methyltransferase activity of SMYD5 increased when the Pol II CTD was added to the in vitro HMT system (Fig. 4f and Supplementary Fig. 5c). The activity was not further changed when the Pol II CTD was phosphorylated. Moreover, to prevent the methyltransferase activity of SMYD5 toward H4K20me3, we used H4K20M octamers as the substrate and detected an increase in methyltransferase activity after the addition of Pol II CTD, whereas the addition of phosphorylated Pol II CTD showed a similar effect (Fig. 4g). Moreover, similar methyltransferase activity was detected when H3K36M mutant octamers were used as the substrate (Supplementary Fig. 5d). Together, these data suggest that the Pol II CTD can bind to SMYD5 to increase its methyltransferase activity toward H3K36me3 and that phosphorylation of the Pol II CTD increases its ability to bind to SMYD5 but not the enzymatic activity of SMYD5.

### The C-terminal glutamic acid-rich domain is important for the methyltransferase activity of SMYD5

**Fig. 5:**
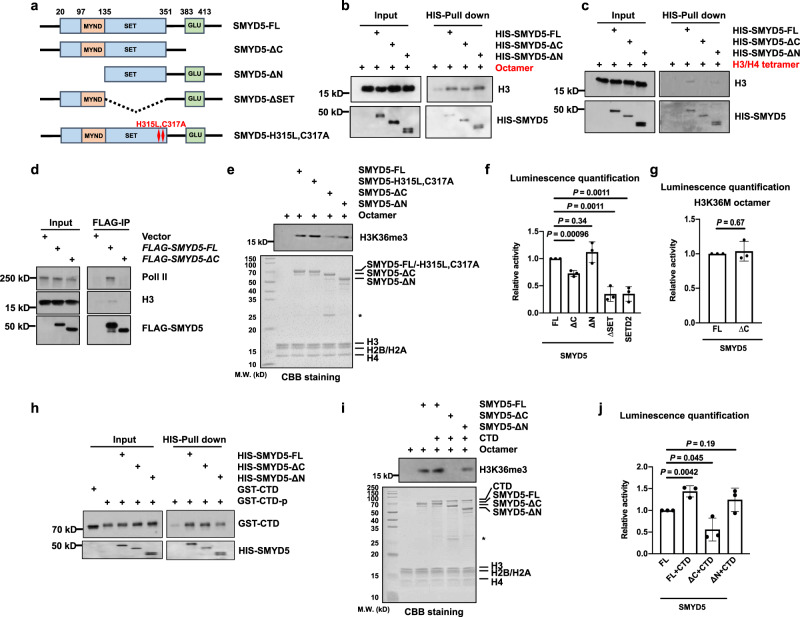
The methyltransferase activity of SMYD5 is regulated by its C-terminal domain. **a** Schema showing the functional domains and mutations of SMYD5. **b** Deletion of the C-terminal domain of SMYD5 reduced its interaction with octamers. Recombinant HIS tagged SMYD5 and SMYD5 mutants were incubated with an equal amount of histone core octamers. NI-NTA Seflnose Resin beads were used to pull down SMYD5. Two independent experiments were performed. **c** Same as in (**b**), except H3/H4 tetramer was used to incubate with WT and mutant SMYD5. Two independent experiments were performed. **d** Removal of the C-terminal domain repressed the interactions among SMYD5, Pol II, and H3. FLAG-tagged SMYD5 full length (FL) or C-terminal deletion (ΔC) over-expressed HEK 293 T were subjected to FLAG IP. Proteins from input and IP samples were analyzed by Western blotting using the indicated antibodies. HEK 293 T transfected with empty vectors were used as the negative controls. Two independent experiments were performed. **e** HMT assays of full length SMYD5 and mutant SMYD5 against octamer substrates. Upper panel, H3K36me3 was detected by Western blotting. Lower panel, input by Coomassie Brilliant Blue (CBB) staining. Each assay was repeated at least three times with similar results. Asterisk indicates non-specific proteins. **f** End-point HMT assays of full length SMYD5 and mutant SMYD5 against core octamers. After the reaction, SAM was transferred to SAH which was detected by the MTase-Glo™ assay. Each assay was repeated at least three times with similar results. *N* = 3 independent experiments. Data are mean ± SD. *P* values were calculated by one-way ANOVA. **g** End-point HMT assays of full length and C-terminal deleted SMYD5 against H3K36M mutant octamers. After the reaction, SAM was transferred to SAH which was detected by the MTase-Glo™ assay. *N* = 3 independent experiments. Data are mean ± SD. *P* values were calculated by one-way ANOVA. **h** N-terminal domain is important for the interaction between SMYD5 and phosphorylated Pol II CTD. GST tagged Pol II CTD was purified and then phosphorylated by the CDK7-Cyclin H complex. Recombinant HIS-tagged full length and mutant SMYD5 were purified and incubated with phosphorylated Pol II CTD, respectively. Ni-NTA Seflnose Resin beads were used to pull down SMYD5. The input and beads-bound proteins were analyzed by Western blotting using the indicated antibodies. Unphosphorylated Pol II CTD was used as the negative control for the mobility shift of phosphorylated Pol II CTD. CTD, Pol II CTD. CTD-p, phosphorylated Pol II CTD. Two independent experiments were performed. **i** HMT assays of full length SMYD5 and mutant SMYD5 against octamer substrates in the presence of Pol II CTD. Upper panel, H3K36me3 was detected by Western blotting. Lower panel, the input of the reactions was shown by Coomassie Brilliant Blue (CBB) staining. Each assay was repeated at least three times with similar results. Star indicated the non-specific proteins. CTD, Pol II CTD. Two independent experiments were performed. **j** End-point HMT assays of full length SMYD5 and mutant SMYD5 against octamer substrates in the presence of Pol II CTD. After the reaction, SAM was transferred to SAH which was detected by the MTase-Glo™ assay. *N* = 3 independent experiments. Data are mean ± SD. *P* values were calculated by one-way ANOVA. CTD, Pol II CTD. Source data are provided as a Source Data file.

SMYD5 has several domains, including the C-terminal glutamic acid rich domain, N-terminal MYND domain, and internal SET domain. In addition, the H315L,C317A mutation was found to abolish its H4K20me3 enzymatic activity^37^ (Fig. 5a). We purified full-length SMYD5 (SMYD5-FL), C-terminal domain-deleted SMYD5 (SMYD5-ΔC), and N-terminal domain-deleted SMYD5 (SMYD5-ΔN) and tested the importance of these domains for the interactions between SMYD5 and octamers. Deletion of the C-terminal domain reduced the binding between SMYD5 and octamers (Fig. 5b). In addition, SMYD5-ΔC exhibited a reduced interaction with H3-H4 tetramers (Fig. 5c). To demonstrate how SMYD5 is regulated in vivo, we overexpressed FLAG-tagged *Smyd5-FL* and *Smyd5-ΔC* in HEK 293 T cells, purified FLAG-tagged SMYD5, and analyzed the co-purified proteins by Western blotting. Pol II and H3 were co-purified with SMYD5-FL but not with SMYD5-ΔC (Fig. 5d), further confirming that the C-terminal domain of SMYD5 is important for the interaction between SMYD5 and H3.

We then conducted an in vitro HMT assay using SMYD5 mutants. Compared with SMYD5-FL, SMYD5-ΔC resulted in lower H3K36me3 levels in octamers. The H315L, C317A and ΔN mutations did not affect the methylated H3K36me3 levels (Fig. 5e). We used a luciferase assay to measure the total enzymatic activity. Only SMYD5-ΔC and SET domain-deleted SMYD5 (SMYD5-ΔSET) showed decreased enzymatic activity (Fig. 5f). Moreover, the enzymatic activity of SMYD5-ΔC toward H3K36M octamers did not differ from that of SMYD5-FL. This result indirectly suggests that deletion of the C-terminal domain didn’t affect the H4K20me3 enzymatic activity of SMYD5 (Fig. 5g).

Because we observed that the Pol II CTD can bind to SMYD5 and increase the enzymatic activity of SMYD5, we sought to determine whether the different domains were important for this process. The unphosphorylated Pol II CTD was used as the control for the mobility shift of the phosphorylated Pol II CTD. Less phosphorylated Pol II CTD was copurified with SMYD5-ΔN than with SMYD5-FL and SMYD5-ΔC, indicating that SMYD5 interacts with the Pol II CTD directly through its N-terminal domain (Fig. 5h). Furthermore, we investigated how the H3K36me3 enzymatic activity of SMYD5 is affected in the presence of the Pol II CTD when the N- or C-terminal domains of SMYD5 is truncated (Fig. 5i, j). While SMYD5-ΔN generated slightly less H3K36me3 than SMYD5-FL in the presence of the Pol II CTD, SMYD5-ΔC showed a strong reduction in H3K36me3. Altogether, these data suggest that SMYD5 binds to the Pol II CTD and histone octamers through its N- and C-terminal domains, respectively, and that deletion of the C-terminal domain largely abolishes its H3K36me3 enzymatic activity.

### Overexpression of *Smyd5* restores enrichment of H3K36me3 at promoters and rescues gene expression in *Smyd5* KO cells

**Fig. 6:**
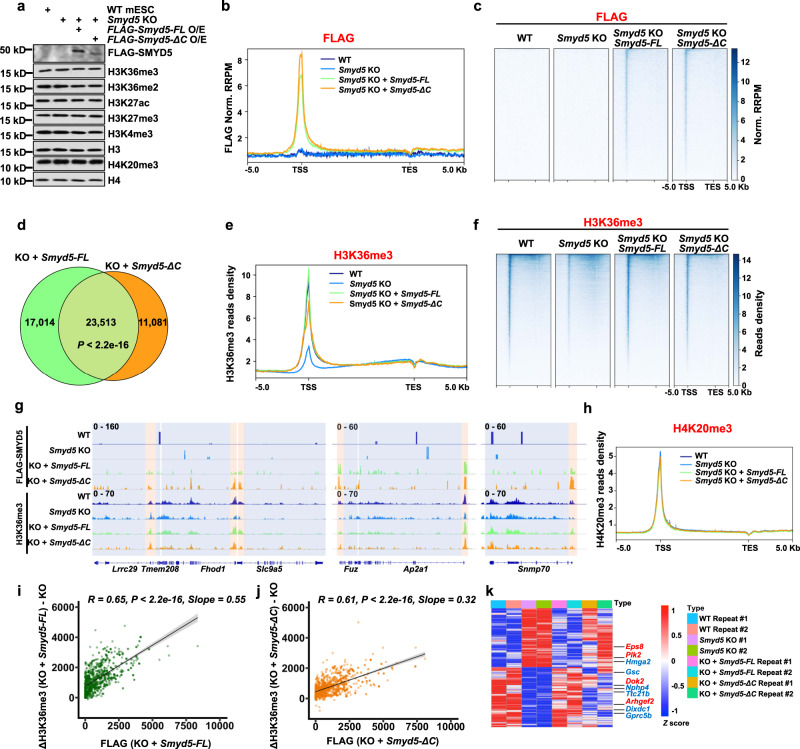
Overexpression of *Smyd5* but not *Smyd5-ΔC* rescues H3K36me3 and gene expression in *Smyd5* KO cells. **a** Overexpression of *Smyd5* in *Smyd5* KO mESCs didn’t change the total levels of tested histone marks. *Smyd5* KO mESCs were transfected with full length (*FLAG-Smyd5-FL*) or C-terminal deletion (*FLAG-Smyd5-ΔC*) *Smyd5* overexpression plasmids. Cell extracts were analyzed by Western blotting using the indicated antibodies. Two independent experiments were performed. Source data are provided as a Source Data file. **b** Normalized read distribution profiles of FLAG-SMYD5 as detected by FLAG CUT&Tag. **c** Heatmaps illustrating FLAG-SMYD5 levels detected by FLAG CUT&Tag around gene body regions. **d** Venn diagram showing the overlap of overexpressed full length and C-terminal deleted SMYD5. *P* value was determined by Fisher’s exact statistical test, two-sided. KO, *Smyd5* KO. **e** Normalized read distribution profiles of H3K36me3 CUT&Tag signals. **f** Heatmaps illustrating H3K36me3 levels around gene body regions. **g** IGV tracks presenting the enrichments of FLAG-SMYD5 and H3K36me3 by CUT&Tag. Three different chromatin loci were shown. Red boxes indicated the promoter regions. Blue boxes indicated gene body regions. **h** The normalized read distribution profiles of H4K20me3 CUT&Tag signals. **i** The difference in normalized read densities of H3K36me3 at promoters between *Smyd5* KO and *Smyd5* reexpression mESCs relative to that of FLAG-SMYD5. R, correlation coefficients that were assessed by Pearson product moment correlation. Confidence interval shows the SEM. Source data are provided as a Source Data file. **j** Same as in (**h**), except *Smyd5* KO mESCs with *FLAG-Smyd5-ΔC* overexpression were used. Confidence interval shows the SEM. Source data are provided as a Source Data file. **k** Gene expression heatmap for genes that were restored in *FLAG-Smyd5-FL* reexpression mESCs. All the changed genes between WT and *Smyd5* KO cells that were ‘rescued’ by the WT *Smyd5* transgene were plotted. Genes associated with Ras signaling were labeled as red. Genes associated with Wnt signaling were labeled as blue. Two repeats of each sequencing were shown. Source data are provided as a Source Data file.

To test whether the changes in H3K36me3 at promoters were caused by *Smyd5* KO, we overexpressed *FLAG*-*Smyd5-FL* and *FLAG-Smyd5-ΔC* in *Smyd5* KO cells. The total levels of H3K36me3 and H4K20me3 were not changed to detectable levels in *Smyd5* KO mESCs or mESCs with *Smyd5* reexpression, as detected by Western blotting (Fig. 6a). The levels of other tested histone marks were not obviously changed. We conducted FLAG CUT&Tag to determine how overexpressed WT and mutant SMYD5 were enriched on chromatin. WT and *Smyd5* KO mESCs were used as the negative control cells for the enrichment of FLAG-tagged proteins. Two independent replicates with high correlations were conducted for each cell line and merged for further analysis (Supplementary Table 1). Similar to endogenous SMYD5, SMYD5-FL and SMYD5-ΔC were enriched at promoters, with SMYD5-ΔC exhibiting higher enrichment (Fig. 6b, c). There were no specific FLAG signals detected in either WT or *Smyd5* KO cells, confirming the specificity of FLAG CUT&Tag. In addition, we identified 40,527 and 34,594 FLAG peaks in *FLAG-Smyd5-FL* and *FLAG-Smyd5-ΔC* reconstituted mESCs, respectively. A total of 23,513 peaks were overlapped significantly between these two cell lines, indicating that SMYD5-FL and SMYD5-ΔC were localized at similar chromatin loci (Fig. 6d).

We further examined the distribution of H3K36me3 when *Smyd5* was reexpressed in *Smyd5* KO mESCs by H3K36me3 CUT&Tag. Two independent replicates with high correlations were conducted and merged for further analysis (Supplementary Table 1). Because the total levels of H3K36me3 were not obviously changed, we didn’t use *E.coli* DNA as the spike-in control to normalize the H3K36me3 signals to avoid over normalization. Overexpression of *FLAG-Smyd5-FL* in *Smyd5* KO mESCs restored enrichment of H3K36me3 at promoters (Fig. 6e, f). Expression of *FLAG-Smyd5-ΔC* also restored H3K36me3 at promoters, albeit to a lower level than did expression of *FLAG-Smyd5-FL*. Since higher enrichment of SMYD5-ΔC than of SMYD5-FL was detected at promoters, this lower re-establishment of H3K36me3 in *FLAG-Smyd5-ΔC* reconstituted cells was unlikely to be due to lower recruitment of the SMYD5 mutant protein to promoters. Furthermore, enrichment of FLAG-tagged SMYD5 and H3K36me3 was revealed at three representative chromatin loci (Fig. 6g). Enrichment of Pol II and H4K20me3 at promoters was not altered in *Smyd5* KO or reconstituted cells (Fig. 6H and Supplementary Fig. 6a–c). To evaluate how H3K36me3 is restored by reexpression of *Smyd5*, we plotted the overexpressed SMYD5 signals and alterations in H3K36me3 at promoters which overlapped with the H3K36me3 peaks in WT cells. We found positive correlations between the changes in H3K36me3 and the SMYD5-FL (R = 0.65) and SMYD5-ΔC (R = 0.61) signals (Fig. 6i, j). In addition, the slope of the simple linear regression line (fit curve) in *FLAG-Smyd5-FL* cells was higher than that in *FLAG-Smyd5-ΔC* cells, supporting the idea that SMYD5-ΔC restores H3K36me3 to a lower level than does SMYD5-FL. Because it’s possible that H3K36me3 CUT&Tag exhibits signals at open chromatin regions, we performed ATAC-seq to elucidate whether the changes of H3K36me3 are correlated with the changes of ATAC-seq in the KO and Smyd5 reconstituted cells. Two replicates of ATAC-seq were performed. The correlations between the changes of H3K36me3 and ATAC-seq signals were quite low and without significances between the WT and KO, *Smyd5*-FL reconstituted and KO, *Smyd5*-ΔC reconstituted and KO cell lines, respectively (Supplementary Fig. 6d–f). This data supports the idea that the H3K36me3 CUT&Tag signals were not from the off-target effect at open chromatin regions. Additionally, we performed RNA-seq in mESCs with reexpression of *Smyd5*. The alterations in gene expression induced by *Smyd5* KO were reversed by transduction of *FLAG-Smyd5-FL* but not *FLAG-Smyd5-ΔC* transduction (similar to the effects of *Smyd5* KO) (Fig. 6k). Moreover, we combined these gene expression data, SMYD5 CUT&Tag and SMYD5 ChIP-seq data to identify the genes directly regulated by SMYD5. The genes which could be restored by WT *Smyd5* expression but not *Smyd5-ΔC* transduction in *Smyd5* KO mESCs were firstly selected. Then these genes were further selected based on the presence of both SMYD5 CUT&Tag and ChIP-seq peaks at their promoters. The selected genes were listed in Supplementary Table 4. Together, these data suggest that overexpression of *Smyd5-FL* but not *Smyd5-ΔC* restores enrichment of H3K36me3 at promoters and rescues gene expressions in *Smyd5* KO cells.

### SMYD5 contributes to tumorigenesis in liver hepatocellular carcinoma

**Fig. 7:**
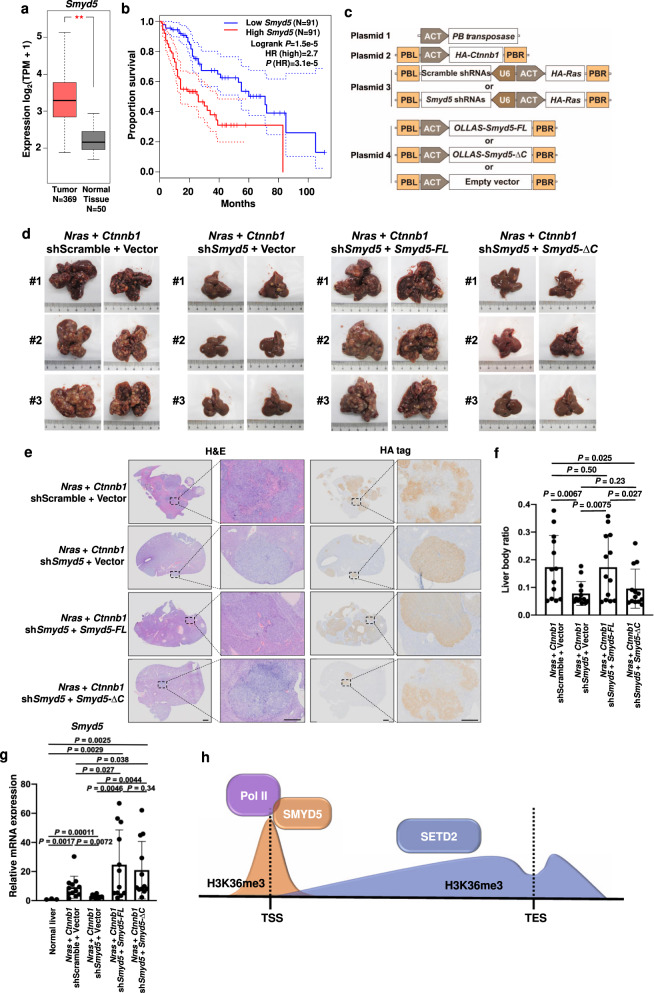
Elevated *Smyd5* promotes the tumorigenesis of liver hepatocellular carcinoma. **a** *Smyd5* was elevated in LIHC tumors compared with normal tissues. The expression levels of *Smyd5* were generated from the TCGA database through GEPIA analysis^43^. TPM, transcript per million. *P* values were calculated by one-way ANOVA. The boxes were drawn from lower quartile (Q1) to upper quartile (Q3) with the middle line denoting the median, and whiskers with maximum 1.5 IQR. **b** Kaplan–Meier analysis of the overall survival in LIHC cases based on *Smyd5* level. Patient data were from the TCGA database through GEPIA analysis^43^. HR, hazard rate ratio. **c** Schema showing the plasmids used for mouse work. ACT, actin promoter. PB, *piggyBac*. PBL and PBR, *piggyBac* repeat termini. U6, U6 promoter. **d** *Smyd5* functions in the tumorigenesis of liver tumors. High expressions of *Nras* and *Ctnnb1* in liver were introduced in mice by HTVI. *Smyd5* was knocked down by shRNAs or reexpressed by overexpression of full length (FL) or C-terminal deletion (∆C) *Smyd5*. Mice were sacrificed 90–100 days after injection and livers were pictured. Three representative livers in each cohort were shown. **e** H&E and HA-tag staining of mouse livers. Mouse livers from different cohorts were fixed, paraffin embedded, sectioned, and stained. CTNNB1 and NRAS were HA-tagged and could be stained by HA-tag staining. Representative livers were shown. Scale bars, 2 mm and 0.5 mm in scanned and zoom in figures, respectively. **f** The liver to body ratio of different cohorts. 13 mice in each group were summarized. Data are mean ± SD. *P* values were calculated by Student’s *t* test, one-sided. Source data are provided as a Source Data file. **g** The expression levels of *Smyd5* were analyzed via RT-PCR. The expression level of *Smyd5* in normal liver tissues from normal mice was set as 1. The data are represented by the mean ± SD. *P* values were calculated by Student’s *t* test, one-sided. Source data are provided as a Source Data file. **h** Schema showing SMYD5 methylates H3K36me3 at promoters and SETD2 is responsible for H3K36me3 in gene bodies. TSS, transcription stat site. TES, transcription end site.

Since SMYD5 is important for the regulation of H3K36me3 and gene expression, we sought to determine whether SMYD5 was altered in tumors by analysis of The Cancer Genome Atlas (TCGA) database. Interestingly, GEPIA analysis showed that *Smyd5* expression was elevated in liver hepatocellular carcinoma (LIHC) tissues compared to normal liver tissues (Fig. 7a)^43^. Furthermore, higher expression levels of *Smyd5* were significantly associated with shortened overall survival times in LIHC (Fig. 7b). We also analyzed the survival rates of patients with high or low *Smyd5* expression in other tumors. The results showed that high expression of *Smyd5* significantly correlated with worse survival only in LIHC. Low expression of *Smyd5* was significantly correlated with worse survival in brain low grade glioma (LGG) (Supplementary Fig. 7a). The expression levels of other histone H3K36 methyltransferases, including *Nsd1*, *Nsd2*, *Nsd3*, *Ash1l*, *Setmar* and *Setd2*, were slightly but nonsignificantly increased in tumors (Supplementary Fig. 7b). In addition, these genes were not associated with a shortened overall survival time in LIHC (Supplementary Fig. 7c). Although the expression of *Setd2* correlated well with that of *Smyd5* in LIHC (Supplementary Fig. 7d), reduced *Setd2* expression was previously reported in mouse liver tumors^44^, suggesting that these two genes lead to liver tumorigenesis in different ways. Moreover, we analyzed the expression of three *Smyd5*-regulated genes— *Ttc21b*, *Nphp4* and *Gpcr5b*—in LIHC (Supplementary Table 4 and Fig. 7e). The expression levels of these genes were highly correlated with the expression level of *Smyd5* (R = 0.5 for *Ttc21b*, R = 0.45 for *Nphp4*, and R = 0.39 for *Gpcr5b*). The expression level of *Smyd5* increased from patients with stage I disease to patients with stage III disease, with a decrease in patients with stage IV disease (Supplementary Fig. 8a). It’s possible that a higher level of *Smyd5* is associated with poorer overall survival so that fewer patients with a high expression level of *Smyd5* survived to develop stage IV disease. Moreover, we analyzed the protein levels of SMYD5 and H3K36me3 in paired non-cancer and cancer tissues from eight patients (Supplementary Fig. 8b). Except one patient (patient #1) who had a lower molecular weight band of SMYD5 in non-cancer tissues, we detected an increase of SMYD5 in cancer tissues than non-cancer tissues. Out of the seven patients with higher SMYD5 in cancer tissues, six patients exhibited higher levels of H3K36me2 in cancer tissues. In mESCs, the increased or decreased protein levels of SMYD5 didn’t change the total levels of H3K36me3 as detected by Western blotting (Fig. 3a). The expressions of SMYD5 and SETD2 were elevated in liver tumors (Supplementary Fig. 7d). These elevated H3K36me3 may not only caused by SMYD5 but also SETD2 in cancer tissues. We then speculated that elevated expression of *Smyd5* is an important marker for LIHC.

There are several pathways/biological processes affected by consensus driver genes in LIHC, including Wnt signaling, chromatin complex, MAPK signaling, cell cycle, and RNA abundance^45^. Our previous GO enrichment analysis results showed that *Smyd5* KO affected the expression of genes associated with Ras signaling, Wnt signaling, and liver development (Supplementary Fig. 4e). In addition, *Ras* and *Ctnnb1* overexpression was able to drive tumorigenesis in LIHC^46^. Therefore, we used hydrodynamic tail vein injection (HTVI) to induce high expression of *Nras* and *Ctnnb1* in the mouse liver. To further demonstrate the function of *Smyd5* in tumor formation, we knocked down *Smyd5* and rescued its expression with *Smyd5-FL* or *Smyd5-ΔC*. Eight shRNAs were designed for knockdown of *Smyd5* and tested in the mouse liver cell line H2.35. Two shRNAs (#3 and #6) showing the highest knockdown efficiency were selected for further experiments (Supplementary Fig. 8c). Four plasmids were used as a Sleeping Beauty system to coexpress *Nras* and *Ctnnb1* together with *Smyd5* shRNAs and *Smyd5* mRNA (Fig. 7c).

Mice were sacrificed 90-100 days after injection, and the livers were imaged (Fig. 7d and Supplementary Fig. 8d). While overexpression of *Nras* and *Ctnnb1* promoted tumorigenesis in the mouse liver, knockdown of *Smyd5* suppressed tumor formation. More importantly, reexpression of *Smyd5-FL* but not *Smyd5-ΔC* after *Smyd5* knockdown restored tumorigenesis. We then examined H&E- and HA-stained liver sections microscopically, confirming the formation of tumors in the liver and the expression of HA-tagged NRAS and CTNNB1 in tumors (Fig. 7e). In line with these observations, the liver-to-body weight ratio was significantly decreased when *Smyd5* was knocked down and was restored by overexpression of *Smyd5-FL* but not *Smyd5-ΔC* (Fig. 7f).

Moreover, we analyzed the expression of *Smyd5* by RT-qPCR. The expression level of *Smyd5* was elevated in *Nras* and *Ctnnb1* induced tumors comparing to livers from untreated mice, whereas *Smyd5* shRNA treatment reduced the expression of *Smyd5* (Fig. 7g). Expression of *Smyd5-FL* and *Smyd5-ΔC* restored the mRNA level of *Smyd5* to a level similar to but slightly higher than that observed without *Smyd5* knockdown. The *Smyd5* levels detected in the reconstituted tumors were the subtotals of the levels of overexpressed and endogenous *Smyd5*, which were depleted and expressed at low levels. The reduced tumor formation in the *Smyd5-ΔC* rescue cohorts of mice was unlikely to be due to the different expression levels of WT and mutant *Smyd5*. Together, these data suggest that SMYD5 is important for liver tumorigenesis of liver tumors and that the effect of SMYD5 is at least partially dependent on its C-terminal domain.

## Discussion

The SET and MYND domain-containing protein family contains five proteins, SMYD1-5^47,48^. SMYD1-3 can catalyze H3K4 methylation whereas SMYD2 and SMYD3 also deposit H3K36me2 and H3K4me1, respectively. The sites of SMYD4-mediated histone methylation are not yet known. SMYD5, which differs from other SMYD proteins in the replacement of the C-terminal tetratricopeptide repeat domain with a distinct polyglutamic acid domain, has been reported to catalyze H4K20me3^37^. However, depletion of both *Suv420H1* and *Suv420H2* in mice largely abolishes H4K20me3, suggesting that SUV420H1/2 are major enzymes responsible for H4K20me3 in cells^49,50^. Our data also show that depletion of *Smyd5* doesn’t affect H4K20me3 (Figs. 2d and 6h). Several lines of evidence have demonstrated the importance of SMYD5 in the regulation of cellular functions. During zebrafish embryogenesis, SMYD5 is indispensable for hematopoiesis^51^. SMYD5-mediated H4K20me3 regulates proinflammatory gene programs^37^. The interaction between SMYD5 and protamine increases the thermal stability of SMYD5 to promote sperm chromatin remodeling^52^. SMYD5 deposits H4K20me3 at heterochromatin regions with repetitive DNA elements to maintain chromosome integrity and maintain mESC self-renewal^53,54^. Here, we uncovered that SMYD5 is recruited by Pol II to catalyze H3K36me3 at promoters. SETD2 is considered the only H3K36me3 methyltransferase, because depletion of SETD2 greatly reduces the total level of H3K36me3 in cells. However, traces of H3K36me3, which are always considered the background signals of antibodies, are still detected by Western blotting^27,28^. Our finding complements to the understanding of how H3K36me3 is regulated (Fig. 7h).

Our data are consistent with previous genome-wide profiling of the H3.3K36me3 reader ZMYND11. ZMYND11 is not localized at promoters when cells are crosslinked before chromatin shearing^20^. Interestingly, strong enrichment of ZMYND11 is detected at promoters in uncrosslinked cells^19^. This differential localization of ZMYND11 in crosslinked and uncrosslinked cells is similar to the distribution of H3K36me3, which is enriched at promoters when cells are not crosslinked during the profiling process.

The C-terminal domain of SMYD5 binds to histone core octamers to regulate the H3K36me3 enzymatic activity of SMYD5. The enriched glutamic acids in the C-terminal domain are negatively charged under physiological conditions, while histones contain many acidic amino acids, which are positively charged. These negative and positive charges can induce interactions between SMYD5 and histone core octamers. Interestingly, the deposition of H4K20me3 is not affected by deletion of the C-terminal domain of SMYD5, which decreases the binding between SMYD5 and histones. We observed that SETD2 methylated H3K36 via a “hit and run” mechanism, leading to a very short retention time for the interaction^39^. It’s possible that the required retention time of SMYD5 and histone contact is longer for H3K36me3 than for H4K20me3. When the interactions between SMYD5 and histones are reduced, the H3K36me3 but not the H4K20me3 enzymatic activity of SMYD5 is inhibited by the shorter retention time. In addition, the N-terminal domain of SMYD5 interacts with the Pol II CTD to increase SMYD5 enzymatic activity. When the C- and N-terminal domain truncations of SMYD5 were expressed, we observed that the Pol II CTD increased the enzymatic activity of N-terminal domain truncated SMYD5 to a lower level than that of WT SMYD5 and that depletion of the C-terminal domain had a strong inhibitory effect in either the presence or absence of the Pol II CTD (Figs. 5i, j). These observations indicate that the interactions between SMYD5 and histones are critical for H3K36me3. The H315L,C317A mutation was first identified from genome sequencing results in patients^37^. In vitro enzymatic activity assays showed that this mutation disrupts the H4K20me3 enzymatic activity of SMYD5. These two amino acid residues were the only sites in the SET domain analyzed, thus, other key amino acid residues may also be important for the enzymatic activity of SMYD5.

When we treated cells with Pol II inhibitors or a high concentration of salt (300 mM NaCl) immediately before tagmentation, enrichment of H3K36me3 at promoters was quickly depleted. It’s unlikely that a demethylase specifically removes the H3K36me3, since these H3K36me3 marks are very dynamic. We speculate that this specific enrichment of H3K36me3 may set histones into a “ready to incorporate” states for incorporation into chromatin. Histones are methylated by SMYD5 before they are deposited onto chromatin. Thus, this process, once inhibited, can lead to the release of histones from chromatin, in turn leading to a quick reduction in H3K36me3 at promoters. Consistent with this idea, SMYD5 favors histone core octamers over DNA-containing nucleosomes as its substrate. The enriched DNA signals in the CUT&Tag and N-ChIP-Rx data likely arose from histones that were ready to be incorporated into chromatin and located very close to chromatin. It would be interesting to investigate whether H3K36me3 at promoters affects the interactions between histones and histone chaperones and, subsequently, the incorporation of histones into chromatin.

The canonical histone H3.1/H3.2 is incorporated into chromatin during S phase. The histone H3 variant H3.3 is deposited into chromatin during gene transcription throughout the cell cycle. When catalyzing H3K36me3, SETD2 preferentially uses H3.3 as the substrate if H3.3S31 is phosphorylated. Via an in vitro HMT assay and structural analysis, Armache et al. found that the enzymatic activities of SETD2 are promoted by H3.3S31ph^55^. Determination of whether SMYD5 preferentially uses H3.1 or H3.3 as the substrate and how H3.3S31ph may affect SMYD5-mediated H3K36me3 would shed light on the regulatory mechanism of SMYD5 enzymatic activity.

Li et al. recently reported that SETD2 deficiency in the liver results in HCC^44^. The reduction in *Setd2* expression modulated the DNA damage response and lipid metabolism in the liver. *Smyd5* regulates the Wnt and/or Ras signaling. These two genes may regulate two different mechanisms during liver tumorigenesis. H3K36me3 at promoters is not totally absent when *Smyd5* is knocked out in mESCs, suggesting that there are other enzymes responsible for enrichment of H3K36me3 at promoters. When we performed screening for H3K36me3 methyltransferases, the target genes were based on the known histone methyltransferases. Other proteins that have not been previously identified as histone methyltransferases could be responsible for this specific enrichment of H3K36me3. An expanded screening library could help to identify undiscovered enzymes. Through years of study, many histone methyltransferases have been found to decorate core histone proteins. An increasing number of the enzymes identified initially have been proven to be responsible for methylating other amino acid residues of histones and even other proteins^50^. A reanalysis of known histone methyltransferases seems warranted.

## Methods

### Cell culture and cell lines

E14 mouse embryonic stem cells (mESCs) were cultured on 0.1% gelatin-coated dishes in DMEM supplemented with 15% fetal bovine serum (FBS), 1% antibiotic solution (penicillin/streptomycin), 1% glutamax, 1% MEM nonessential amino acids, 1% sodium pyruvate, 0.1 mM-mercaptoethanol and 1000 U/ml recombinant leukemia inhibitory factor (LIF). HEK 293 T cells and NIH3T3 cells were cultured in DMEM supplemented with 1% glutamax, 10% FBS and 1% antibiotic solution (penicillin/streptomycin). H2.35 cells were cultured in DMEM supplemented with 1% glutamax, 200 nM Dexamethasone, and 4% FBS. All cells were grown at 37 °C and 5% CO_2_. K562 cells were cultured in RPMI1640 supplemented with 1% glutamax, 1% antibiotic solution (penicillin/streptomycin) and 10% FBS.

mESCs KO cell lines for screening, including *Setd2*, *Smyd5* KO, were constructed by sgRNAs, which were cloned into pSpCas9(BB)-2A-Puro from Dr. Feng Zhang (Addgene plasmid #48139).

### Cell proliferation and alkaline phosphatase staining

mESCs were seeded into 96 well plates in growth medium for 0 h, 24 h, 48 h, 72 h, and 96 h, respectively. CellTiter-Blue kit (Promega, Cat. #G8081) was used to measure cell viability at different time points. Fluorometric signals were recorded by a microplate reader (BioTek, Synergy NEO2).

mESCs were fixed with 1% paraformaldehyde before alkaline phosphatase staining. The activity of alkaline phosphatases in mESCs was detected by INT/BCIP (Brown) kit (Sangon Biotech, Cat. #C500033) following the manufacturer’s instructions. NIH3T3 cells were used as negative controls.

### ChIP-seq

Cells for X-ChIP-Rx were crosslinked with 1% paraformaldehyde for 10 min and then quenched in 125 mM glycine for 5 min at room temperature with rotation, while cells for N-ChIP-Rx were processed without paraformaldehyde crosslink. After washed with cold PBS, cells were lysed in lysis buffer (10 mM Tris-HCl, pH 7.5, 10 mM NaCl, and 0.5% NP-40) on ice for 10 min. The nuclei were spun down and then digested in 500 µl MNase digestion buffer (20 mM Tris-HCl, pH 7.5, 15 mM NaCl, 60 mM KCl, and 1 mM CaCl_2_) with 1000 units of MNase (NEB, Cat. #M0247S) by incubating at 37 °C for 20 min. The reaction was stopped by adding 2X STOP buffer (100 mM Tris-HCl, pH 8.1, 20 mM EDTA, 200 mM NaCl, 2% Triton X-100, and 0.2% sodium deoxycholate). Sonication was performed in X-ChIP-Rx, but not N-ChIP-Rx, for 5 cycles of 30 s on and 30 s off in Bioruptor. After centrifuged at 15,000 × *g* at 4 °C for 10 min, the supernatant was used for chromatin immunoprecipitation. Same amounts of Drosophila S2 cell chromatin were spiked in for normalization. 10 µg of H3K36me3 antibodies (Abcam, Cat. #ab9050) or IgG was added into each sample and rocked at 4 °C overnight. Protein G beads were added and incubated for 3 h. After the beads were extensively washed, the bound DNA was eluted by elution buffer (10 mM Tris-HCl, pH 8.0, 10 mM EDTA, 150 mM NaCl, 5 mM DTT, and 1% SDS). Reverse-crosslinked at 65 °C overnight. The eluted DNA was treated with proteinase K and RNase A, and then purified with the Min-Elute PCR purification kit (Qiagen, Cat. #28006). Chromatin contents were measured by Qubit assay (Vazyme, Cat. #EQ121-02-AA), and subjected to library preparation with TruePrep DNA library prep kit (Vazyme, Cat. #TD501-01) following the manufacturer’s instructions. The library was sequenced on an Illumina NovaSeq platform with pair-end reads of 150 bp.

### CUT&Tag

CUT&Tag was performed as previously described^30^. In brief, 10^5^ cells were harvested in NE buffer (20 mM HEPES-KOH, pH 7.5, 0.5 mM spermidine, 10 mM KCl, 0.1% TritonX-100, 10% Glycerol, 1 mM PMSF) and on ice for 10 min. ConA beads were pre-washed and re-suspended by binding buffer (20 mM HEPES-KOH, pH 7.5, 10 mM KCl, 1 mM CaCl_2_, 1 mM MnCl_2_). 10 µl beads were added to each sample and incubated at room temperature for 10 min. The beads were washed with washing buffer (20 mM HEPES-KOH, pH 7.5, 0.5 mM spermidine, 150 mM NaCl, 0.1% BSA) and resuspended in blocking buffer (20 mM HEPES-KOH, pH 7.5, 0.5 mM spermidine, 150 mM NaCl, 0.1% BSA, 2 mM EDTA) at room temperature for 5 min. Primary antibodies were added by 1:100 dilution and incubated at room temperature for 2 h. After washed with washing buffer, secondary antibodies were added by 1:100 dilution and incubated at room temperature for 30 min. 1.2 µl PA-Tn5 transposomes were added to each sample and incubated at room temperature for 30 min. After washed with washing buffer, if indicated, beads were treated with Pol II inhibitors or high salt (300 mM NaCl) in washing buffer for 10 min. Beads were resuspended in 30 µl washing buffer with 10 mM MgCl_2_ and incubated at 37 °C for 1 h. Reactions were stopped by adding 5.5 µl stop buffer (2.25 μl of 0.5 M EDTA, 2.75 μl of 10% SDS and 0.5 μl of 20 mg/ml Proteinase K) and incubated at 55 °C for 30 min, and then 70 °C for 20 min to inactivate Proteinase K. 0.9X of VAHTS DNA clean beads (VAHTS, Cat. #N411-03) were added to each sample to extract the tagmentated DNA.

Primary antibodies were Rabbit polyclonal anti-Histone H3K36me3 (Active Motif, Cat. #61101) if indicated; H3K36me3 antibodies (Abcam, Cat. #ab9050); Mouse monoclonal anti-RNA polymerase II (Millipore, Cat. #05-623); Mouse monoclonal anti-FLAG M2 (Sigma, Cat. #F1804); Rabbit polyclonal anti-SMYD5 (this study).

### Screening of histone methyltransferases

sgRNAs were designed for each picked gene and single clones were generated by the sgRNA with CRISPR/Cas9 knockout. The KO clones were tested for knockout by Sanger sequencing and then subjected to H3K36me3 CUT&Tag to test whether H3K36me3 at promoter regions were decreased. After the first screening, an independent sgRNA was designed for each pick gene and single clones were generated by this new sgRNA. The KO of genes were identified by Sanger sequencing and cells were subjected to H3K36me3 CUT&Tag in the first screening.

### Octamer and nucleosome reconstitutions

Histone extraction was performed as previously described^56^. Plasmids carried histone proteins, pRSFDuet1-hH3.3/H4 and pRSFDuet1-hH2A/H2B which were kind gifts from Dr. Ruiming Xu were used. For the nucleosome construction, the ‘601’ 167 bp DNA and histone octamers were titrated at a molar ratio of 0.9:1, and mixed together in initiation buffer (2 M NaCl, 10 mM Tris, pH 7.5, 1 mM EDTA, and 1 mM DTT). The mixture was step-wisely dialyzed to the final buffer (0.2 M NaCl, 10 mM Tris, pH 7.5, 1 mM EDTA, and 1 mM DTT) at 4 °C for 36 h. The final mixture included nucleosomes and a small proportion of free DNA. The fractions containing mononucleosomes were collected by gel filtration and further loaded to centrifugal filters for purification and concentration.

### Protein purification

The wild-type and mutant *Smyd5* cDNAs were cloned into the GST-tagged pGEX-6p-1 vector and HIS-tagged pET28a-smt3 vector, respectively. The CTD domain of RNA Pol II in the GST-tagged pGEX-6p-1 vector was a kind gift from Dr. Huasong Lu. Briefly, the plasmids were transformed into *E.coli* BL21 cells (Weidi Biotechnology) and grew to OD600 at around 0.6. 0.1 mM isopropyl β-d-1-thiogalactopyranoside (IPTG) was added to induce the expression of recombinant proteins. The lysates were bound to GST (Sangon Biotech, Cat. #C600031) or NI-NTA Seflnose Resin beads (Sangon Biotech, Cat. #C600033) at 4 °C for 2–4 h. The beads were extensively washed and beads-bound proteins were eluted by 100 mM L-glutathione or 250 mM imidazole respectively. The eluted proteins were dialyzed to storage buffer (40 mM Tris, pH 8.0, 150 mM NaCl, 5% glycerol for GST-tagged proteins, or 10% glycerol in TBS for HIS-tagged proteins) before usage.

### Phosphorylation of CTD

Plasmid pRK5M-Flag-CDK7/cyclin H and pRK5M-Flag-CDK9/cyclin T1 which were kind gifts from Dr. Huasong Lu were co-transfected into HEK 293 T cells respectively. After 48–72 h, cells were harvested and lysed in whole-cell extraction buffer (50 mM HEPES, pH 7.9, 350 mM NaCl, 1% NP-40, 5 mM EDTA, 0.1 mM PMSF) at 4 °C for 20 min. The supernatant was collected after high-speed spinning. FLAG M2 agarose beads (Sigma, Cat. #M8823) were added into supernatant to bind CDK7/cyclin H or CDK9/cyclin T1 complex at 4 °C for 3 h. Beads were extensively washed in washing buffer (20 mM HEPES, pH 7.9, 15% Glycerol, 100 mM KCl, 0.2 mM EDTA, 0.1% NP-40) 5 times and stored in PBS at 4 °C.

The kinase reaction was performed in BF buffer (10 mM MgCl_2_, 50 mM NaCl, 50 mM HEPES, pH 7.4) by adding 2 µg CTD protein, 2 µl of 1 mM ATP, and 10 µl kinase-bound beads for 25 µl mixture in total. The reaction was incubated at 30 °C for 1 h. After high-speed spinning, the supernatant containing phosphorylated CTD protein was saved for the Pull-down assay immediately.

### Primers

Primers used in this study were listed in Supplementary Table 5.

### In vitro Pull-down assay

For the octamer and CTD domain pull down assay, wild-type or mutant human SMYD5 proteins with HIS tag were added into TBS buffer containing 1 µg GST-CTD protein or 1 µg octamers and incubated at 4 °C for 2 h. The pre-washed NI-NTA Seflnose Resin beads (Sangon Biotech, Cat. #C600033) were added for HIS-Pull down. The pre-washed GST Seflnose Resin beads (Sangon Biotech, Cat. #C600031) were added for GST-pull down. Beads were added into the binding reaction and incubated at 4 °C for 4 h in a rotisserie-style tube rotator. The beads were extensively washed by TBS, and boiled with SDS loading buffer before being analyzed by Western blotting.

### In vitro histone methyltransferase assay

Histone methyltransferase reaction was performed in HMT buffer (0.25 M Tris-HCl, pH 8.5, 12.5 mM MgCl_2_, 2.5 mM DTT). 1 µg reconstituted histone octamers or nucleosomes were used as the substrates, 0.5 µl S-adenosylmethionine (NEB, Cat. #B9003) was used as the methyl-donor, and 1 µg purified proteins were added as methyltransferases. The reaction was carried out at 30 °C for 1 h and stopped by adding SDS loading buffer. Samples were analyzed by Western blotting or Coomassie Brilliant Blue staining.

For the MTase-Glo™ assay, after the HMT reaction was completed, the MTase-Glo™ Reagent (Promega, Cat. #V7602) was added to convert SAH to ADP at room temperature for 30 min. And then the detection solution was added to convert ADP to ATP which was detected via a luciferase reaction. The Luminescence was detected by a microplate reader (BioTek, Synergy NEO2). The enzymatic activity of each substrate was normalized to its own negative control (reaction mix without incubation for HMT).

### Mass spectrometry analysis

The purified proteins after histone methyltransferase reaction were analyzed on an Xevo G2-XS QTOF MS System (Waters Corporation) equipped with an electrospray ionization (ESI) source in conjunction with Waters ACQUITY UPLC I-Class plus. Separation and desalting were carried out on a Waters ACQUITY UPLC Protein BEH C4 Column (300 Å, 2.1 × 50 mm, 1.7 μm). Mobile phase A was 0.1% formic acid in water and mobile phase B was acetonitrile with 0.1% formic acid. A constant flow rate of 0.2 ml/min was used. Data was analyzed using Waters UNIFI software. Mass spectral deconvolution was performed using UNIFI software (version 1.9.4, Waters Corporation).

### Immunoprecipitation

Human wild-type and mutant *Smyd5* were cloned into the pcDNA3.1-FLAG plasmid to be overexpressed in HEK293T cells for immunoprecipitation. The transfected HEK293T cells were lysed in IP buffer (50 mM HEPES, pH 7.4, 200 mM NaCl, 0.5% NP-40, 10% Glycerol, 1 mM EDTA). After spun at 15,000 x *g* 4 °C for 10 min, the supernatant was used to bind with FLAG M2 agarose beads (Sigma, Cat. #M8823) at 4 °C overnight. The beads were extensively washed by washing buffer (50 mM HEPES, pH 7.4, 100 mM NaCl, 0.01% NP-40, 10% Glycerol, 1 mM EDTA). Beads-bound proteins were eluted by boiling with SDS loading buffer and then analyzed by Western blotting.

For endogenous protein immunoprecipitation, mouse monoclonal anti-RNA polymerase II (Millipore, Cat. #05-623) or IgG was added to the lysed samples and incubated at 4 °C overnight. Sheep-anti-mouse IgG Dynabeads (Thermo, Cat. #11201D) were added into the samples and incubated at 4 °C for 2 h. After being extensively washed with washing buffer (50 mM HEPES, pH 7 .4, 100 mM NaCl, 0.01% NP-40, 10% Glycerol, 1 mM EDTA), beads-bound proteins were eluted by boiling with SDS loading buffer. Eluted proteins were subjected to Western blotting analysis.

### Global run-on sequencing

The Global run-on sequencing (GRO-seq) was performed as previously described^57^. In brief, 10^7^ cells were used for nuclei isolation per sample. Nuclear Run-On was performed at 37 °C for 5 min. Brominated UTP was incorporated in the Nascent RNA during this step. RNA was extracted by Trizol reagent (Sangon Biotech, Cat. #B610409) and immunoprecipitated with mouse monoclonal anti-BrdU antibody (Abcam, Cat. #ab8955) and sheep-anti-mouse IgG Dynabeads (Thermo, Cat. #11201D). The Purified nascent RNA was used for library preparation by RNA-seq library prep kit (VAHTS, Cat. #NR604).

### Immunofluorescence

Cells were seeded on coverslips into 24 well plate and washed with 1 ml PBS three times. Cells were fixed with 4% PFA for 10 min, then washed with 1 ml PBS twice. Permeabilization was performed in 0.5% Triton X-100 solution with 5% normal goat serum for 1 h at room temperature. Cells were washed with PBS twice. Primary antibodies were diluted 1: 100 in PBS, 4 °C overnight. After washed with 1 mL PBS-T (1X PBS with 0.1% Tween 20) for 3 times, 1:1000 diluted with PBS, Alexa fluor 488 antibodies were added for 1 h in dark at room temperature. After washed with 1 mL PBS-T for 3 times in dark, 5 min each, 1:1000 diluted DAPI (Invitrogen, Cat. #D1306) in PBS was added for 5 min in dark. Cells were washed with PBST twice. Slides were sealed with mounting medium, and then analyzed under fluorescence microscope (Zeiss, LSM 880).

### Animal model

The hydrodynamic tail-vein injection was performed as described previously^58^. Liver tumors were induced by transposon-mediated integration and expression of HA-tagged oncogenes *Nras* and *Ctnnb1*. For *Smyd5* knockdown, two shRNAs were designed and efficiency was validated in mouse liver cell line H2.35. shRNAs were then expressed by the U6 promoter in tandem with *Ras*. For *Smyd5* reexpression, OLLAS-tagged mouse *Smyd5* was expressed by ACT promoter in an individual transposon plasmid. Plasmids used for hydrodynamic tail-vein injection were prepared by Qiagen EndoFreeMaxi Kit, and were dissolved in sterile Ringer’s solution (5.6 mM KCl, 154 mM NaCl, 2.2 mM CaCl_2_, 2.4 mM NaHCO_3_) equal to 10% mice body weight. 62.5 μg of total transposon plasmids and 17 μg *piggyBac* transposase plasmids were delivered through the tail vein by hydrodynamic injection. Mice were sacrificed 90–100 days after injection. Livers were pictured, weighted, and fixed or snap-frozen in liquid nitrogen for further usage. In this study, four-week-old male specific-pathogen-free ICR mice were purchased by Shanghai SLAC Laboratory Animal Company, and kept in SPF facilities at Zhejiang University Laboratory Animal Center. All the mice study methods were approved by Zhejiang University Animal Care and Use Committee.

### Hematoxylin-Eosin staining

The paraffin slides were deparaffined and rehydrated by xylene and alcohol gradients. Hematoxylin-Eosin staining was carried out by HE staining Kit (Sangon Biotech, Cat. #E607318) following the manufacturer’s instructions. The slides were stained with Hematoxylin staining solution for 5 min, differentiated with 0.1% hydrochloric acid-ethanol for 10 s, and counterstained in Eosin staining solution for 30 s. Slides were dehydrated in alcohol and xylene gradients and then sealed with a mounting medium. The slides were analyzed and scanned under microscopy (OLYMPUS, VS120).

### Immunohistochemistry staining

Immunohistochemistry (IHC) staining of paraffin sections was performed by the avidin-biotin complex (ABC) method as previously described^59^. In brief, the paraffin slides were deparaffined and rehydrate by xylene and alcohol gradients. Antigen retrieval was done by heating in citrate buffer, pH 6. Endogenous peroxidase activity was quenched by incubating slides in 2% H_2_O_2_/distilled H_2_O for 30 min. the slides were blocked in 10% goat normal serum with Avidin. Primary Rabbit monoclonal anti-HA-tag antibody (Cell Signaling Technology, Cat. #3724) was added by 1:200 dilution with biotin and incubated at 4 °C overnight. A biotinylated secondary antibody was added by a 1:200 dilution and incubated at room temperature for 30 min. After washed with PBS 3 times, the slides were incubated with VECTASTAIN elite ABC reagent for 30 min. Slides were stained with peroxidase substrate solution, DAB (Sangon Biotech, Cat. #E670033), for 2–10 min until desired staining intensity was developed, and then stopped with PBS. In addition, slides were stained with hematoxylin, de-stained with hydrochloric acid-ethanol, and reversed blue in ammonium solution. Slides were dehydrated in alcohol and xylene gradients, sealed with mounting medium, and then analyzed under microscopy (OLYMPUS, VS120).

### Reverse transcription and qPCR

The normal livers and tumors were ground with steel balls, and RNA was extracted by Trizol reagent (Sangon Biotech, Cat. #B610409). cDNA was then synthesized by One-step gDNA Removal and cDNA synthesis SuperMix kit (TransGen, Cat. #AE311) following the manufacturer’s instructions.

Quantitative RT-PCR was performed using Hieff qPCR SYBR Green Master Mix (YEASEN, Cat. #11201ES08) with Quanta gene q225 qPCR system (Kubo Technology, Beijing). The expression levels of *Sdha* were used as the control to normalize the expressions of target genes. Primers used were listed in Supplementary Table 5.

### Antibodies used in the study

Rabbit polyclonal anti-Histone H3 (Abcam, Cat. #ab1791), 1:5000 diluted for Western blot; Rabbit monoclonal anti-Histone H3K36me3 (Cell Signaling Technology, Cat. #4909, Clone name D5A7, Lot 5), 1:1000 diluted for Western blot; Rabbit polyclonal anti-Histone H3K4me3 (Abcam, Cat. #ab8580, Lot GR3425199-1), 1:1000 diluted for Western blot; Rabbit polyclonal anti-Histone H3K9me3 (Abcam, Cat. #ab8898, Lot GR3291043-1), 1:100 diluted for CUT&Tag; Rabbit monoclonal anti-Histone H3K27me3 (Cell signaling Technology, Cat. #9733, Clone name C36B11, Lot 19), 1:1000 diluted for Western blot, 1:100 diluted for CUT&Tag; Rabbit polyclonal anti-Histone H3K27ac (Abcam, Cat. #ab4729), 1:1000 diluted for Western blot, 1:100 diluted for CUT&Tag; Rabbit monoclonal anti-SETD2 (Abcam, Cat. #ab239350, Clone name EPR20927-67), 1:1000 diluted for Western blot; Rabbit polyclonal anti-Histone H4K20me3 (Abcam, Cat. #ab9053), 1:1000 diluted for Western blot; Mouse monoclonal anti-Histone H4 (Abcam, Cat. #ab31830, Clone name mAbcam 31830, Lot GR3204774-5), 1:1000 diluted for Western blot; Mouse monoclonal anti-BrdU (Abcam, Cat. #ab8955, Clone name IIB5, Lot GR3362257-4), 1:100 diluted for GRO-seq; Mouse monoclonal anti-FLAG M2 (Sigma, Cat. #F1804, Clone name M2), 1:1000 diluted for Western blot, 1:100 diluted for CUT&Tag; Rabbit polyclonal anti-RNA polymerase II CTD phospho S2 (Abcam, Cat. #ab5095, Lot GR3225147-1), 1:1000 diluted for Western blot; Mouse monoclonal anti-RNA polymerase II CTD phospho S5 (Abcam, Cat. #ab5408, Clone name 4H8, Lot GR3264297-4), 1:1000 diluted for Western blot; Mouse monoclonal anti-RNA polymerase II (Millipore, Cat. #05-623, Clone name CTD4H8, Lot 3489764), 1:1000 diluted for Western blot, 1:100 diluted for CUT&Tag; Rabbit polyclonal anti-Histone H3K36me3 (Active Motif, Cat. #61101, Lot 28818005), 1:100 diluted for CUT&Tag; Rabbit polyclonal anti-Histone H3K36me3 (Abcam, Cat. #ab9050, Lot GR3382010-2), 1:100 diluted for CUT&Tag, 10 µg for ChIP-seq; Mouse monoclonal anti-GST Tag (Sangon Biotech, Cat. #D190101, Lot G112AA0001), 1:1000 diluted for Western blot; Mouse monoclonal anti-6x HIS Tag (Sangon Biotech, Cat. #D191001, Lot GB15AA0001), 1:1000 diluted for Western blot; Rabbit monoclonal anti-HA-tag antibody (Cell Signaling Technology, Cat. #3724, Clone name C29F4), 1:200 diluted for IHC; Rabbit polyclonal anti-SMYD5 (this study); Peroxidase AffiniPure Goat anti-Rabbit IgG (H + L) (Jackson ImmunoResearch Laboratories, Cat. #111-035-003, Lot 158673), 1:5000 diluted for Western blot. Alexa Fluor^®^ 488 AffiniPure Alpaca Anti-Rabbit IgG (H + L) (Jackson ImmunoResearch Laboratories, Cat. #611-035-215), 1:1000 diluted for IF.

### Epigenomic data processing and visualization

To remove adaptors and low-quality reads, Trim Galore (version 0.6.4) (https://www.bioinformatics.babraham.ac.uk/projects/trim_galore [https://www.bioinformatics.babraham.ac.uk/projects/trim_galore]) was used with the parameter ‘–paired’. Trimmed reads were then mapped to the mouse reference genome mm9 using Bowtie2^60^ (version 2.3.5.1). PCR duplicates were removed by GATK4 (version 4.1.4.0) (https://github.com/broadinstitute/gatk [https://github.com/broadinstitute/gatk]) with the parameter ‘–REMOVE_DUPLICATES = true’ in ChIP-seq data while not removed in the CUT&Tag. MACS2^61^ (version 2.2.6) was used to call broad peaks for H3K36me3. Specially, FLAG-SMYD5 peaks were called by SEACR^62^ (version 1.3) for sparse enrichment analysis.

For visualizing epigenomic signals, normalizing mapped reads, and calculating the coverage of features across the genome, BEDtools^63^ (version 2.92.2) and bedGraphToBigWig approach (version 4) (https://www.encodeproject.org/software/bedgraphtobigwig/ [https://www.encodeproject.org/software/bedgraphtobigwig/]) were used with the following parameters ‘genomecov–scaleFactor 10,000,00/(the number of reads mapped to *E.coli, S.pombe* or *D. melanogaster* genome)’. deepTools^64^ (version 3.4.3) was used to draw heatmaps by function computeMatrix and plotHeatmap. Normalized signals were visualized in Integrative Genomics Viewer (IGV)^65^ (version 2.6.3). Peak distribution and annotation were generated by ChIPseeker^66^ (version 1.22.1). For downstream differential peaks analysis, multiBigwigSummary in deepTools was used to count average signals over each region. Differential regions analysis was performed by limma^67^ (version 3.42.2) with normalized signals. clusterProfiler^68^ (version 3.14.3) was used to perform GO analysis. The correlations between two repeats were summarized in Supplementary Table 1. The correlations were calculated by the Pearson product moment correlation. The Venn plots showing the overlap of peaks were performed by ChIPpeakAnno^69^ (version 3.26.2).

### CUT&Tag screening

Promoter regions were defined as 500 bp around TSS. Gene body regions were defined as 500 bp downstream of TSS to TES. To calculate the read density at promoters and gene body regions, computeMatrix was used with scale-regions mode. H3K36me3 index, defined as Index = $\frac{{{{\mbox{normalized}}}}\; {{{\mbox{signals}}}}\; {{{\mbox{at}}}}\; {{{\mbox{TSS}}}}\pm 500{{{{{\rm{bp}}}}}}}{{{{\mbox{normalized}}}}\; {{{\mbox{signals}}}}\; {{{\mbox{at}}}}\; {{{\mbox{Gene}}}}\; {{{\mbox{Body}}}}}$, was used as an indicator for screening.

### RNA-seq data processing and analysis

Trim Galore was used to remove sequencing reads with adaptors or low-quality bases with the parameter ‘–paired’. STAR^70^ (version 2.5.4b) was used for mapping the filtered reads to mouse reference genome mm9. Expression results were counted using featureCounts^71^ (version 2.0.0) and normalized as reads per kilobase per million mapped reads (RPKM) by edgeR^72^ (version 3.28.1). edgeR was used to find genes with different expression levels, which were selected by the threshold abs(log_2_(*foldchange*)) > 0.5 and *P* < 0.05. The volcano plot showing differential genes was generated by ggplot2^73^ (version 3.3.2).

## Supplementary information

Supplementary Information

## Data Availability

The data that support this study are available from the corresponding author upon reasonable request. The reference genome used in this study is available in UCSC database (http://genome.ucsc.edu) under mouse reference genome mm9. The raw and processed sequencing data generated in this study have been deposited in the NCBI Gene Expression Omnibus (GEO) database under accession code GSE184894. ENCODE [https://www.encodeproject.org/] datasets were downloaded with the accession numbers: H3K36me3 (ENCSR000CGR). The other external datasets were downloaded from NCBI Gene Expression Omnibus (GEO) [http://www.ncbi.nlm.nih.gov/geo/], with the accession numbers: GSE169049 and GSE94086. Mass spectrometry data were deposited into the MassIVE repository with accession number MSV000089303. Source data are provided with this paper.

## Source data

Source Data
