# Supplementary material for: SMYD5 catalyzes histone H3 lysine 36 trimethylation at promoters
Source: Nat Commun. 2022 Jun 9;13:3190. doi: 10.1038/s41467-022-30940-1 (PMC9184575; doi:10.1038/s41467-022-30940-1)
Supplement: Supplementary file 1 — Supplementary Information [file 41467_2022_30940_MOESM1_ESM.pdf]

SMYD5 catalyzes histone H3 lysine 36 trimethylation at promoters

Yanjun Zhang[1, #], Yuan Fang[1, #], Yin Tang[1, #], Shixun Han[1], Junqi Jia[1], Xinyi Wan[1], Jiaqi Chen[2], Ying Yuan[2], Bin Zhao[1], and Dong Fang[1, 2, *]

[1] Zhejiang Provincial Key Laboratory for Cancer Molecular Cell Biology, Life Sciences Institute, Zhejiang University, Hangzhou, Zhejiang 310058, China.

[2] Department of Medical Oncology, Key Laboratory of Cancer Prevention and Intervention, Ministry of Education, The Second Affiliated Hospital, Zhejiang University School of Medicine, Hangzhou, Zhejiang, China.

[#] These authors contribute equally

[*] Correspondence: dfang@zju.edu.cn

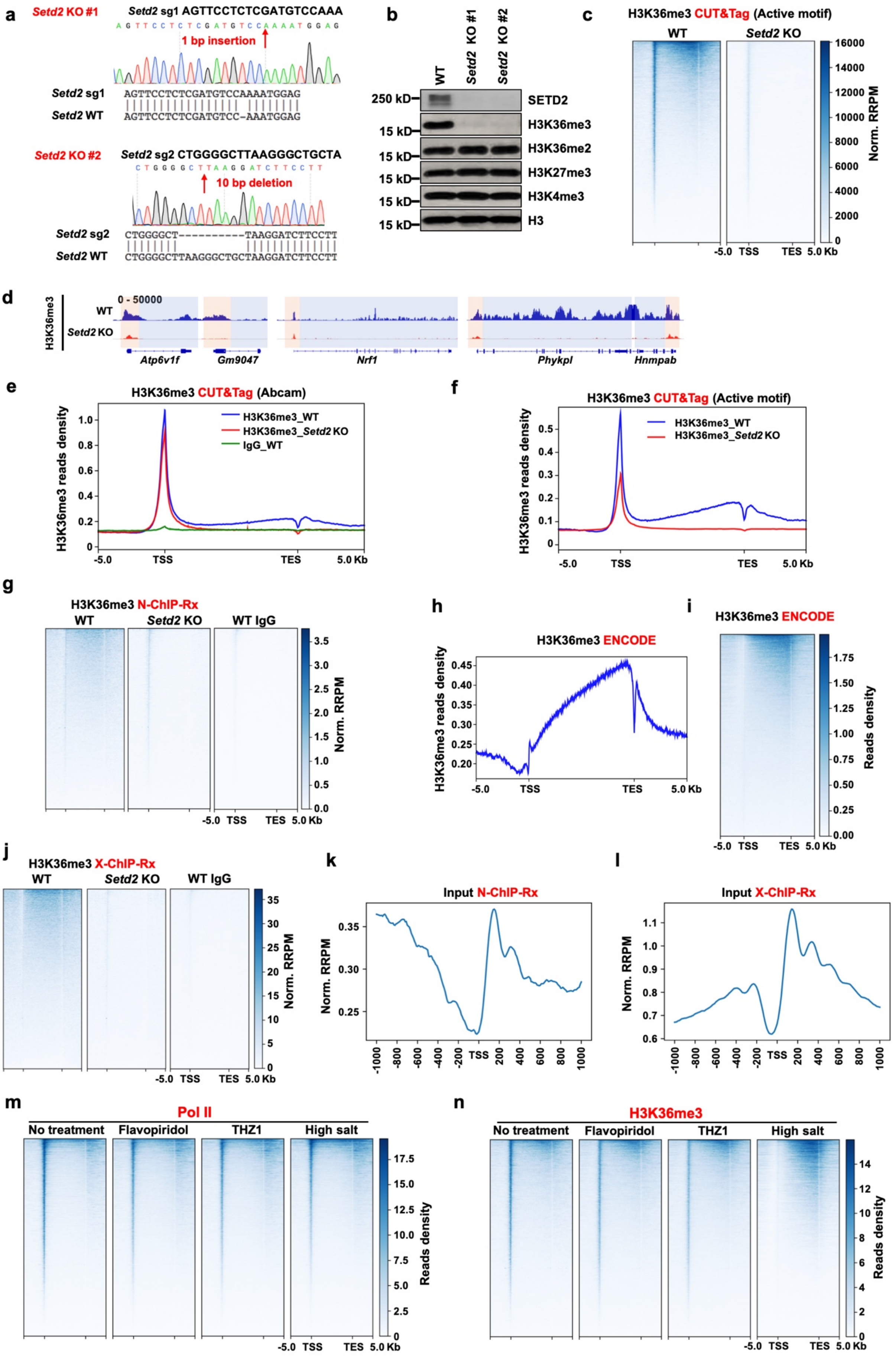
a
Setd2 KO #1
Setd2 sg1 AGTTCCTCTCGATGTCCAAA
1 bp insertion
Setd2 sg1 AGTTCCTCTCGATGTCCAAAATGGAG
Setd2 WT AGTTCCTCTCGATGTCC-AAATGGAG
Setd2 KO #2
Setd2 sg2 CTGGGGCTTAAGGGCTGCTA
10 bp deletion
Setd2 sg2 CTGGGGCT----------TAAGGATCTTCCTT
Setd2 WT CTGGGGCTTAAGGGCTGCTAAGGATCTTCCTT
b
WT
Setd2 KO #1
Setd2 KO #2
250 kD
15 kD
SETD2
H3K36me3
H3K36me2
H3K27me3
H3K4me3
H3
c
H3K36me3 CUT&Tag (Active motif)
WT
Setd2 KO
Norm. RRPM
-5.0 TSS TES 5.0 Kb
d
H3K36me3
0 - 50000
WT
Setd2 KO
Atp6v1f
Gm9047
Nrf1
Phykpl
Hnmpab
e
H3K36me3 CUT&Tag (Abcam)
H3K36me3_WT
H3K36me3_Setd2 KO
IgG_WT
H3K36me3 reads density
f
H3K36me3 CUT&Tag (Active motif)
H3K36me3_WT
H3K36me3_Setd2 KO
g
H3K36me3 N-ChIP-Rx
WT
Setd2 KO
WT IgG
h
H3K36me3 ENCODE
i
H3K36me3 ENCODE
Reads density
j
H3K36me3 X-ChIP-Rx
k
Input N-ChIP-Rx
l
Input X-ChIP-Rx
m
Pol II
No treatment
Flavopiridol
THZ1
High salt
n
H3K36me3

Supplementary Figure 1. H3K36me3 is localized at the promoters in mESCs.

(**a**) Two alleles of *Setd2* were mutated in KO clones. The Sanger sequencing results of the *Setd2* gene locus were shown. 1 bp insertion and 10 bp deletion were identified at both alleles in *Setd2* KO #1 and #2 clones, respectively.

(**b**) Western blotting result showing the levels of indicated proteins in WT and *Setd2* KO mESCs. Cell extracts were analyzed by Western blotting using the specified antibodies. Two independent experiments were performed. Source data are provided as a Source Data file.

(**c**) Heatmaps illustrating H3K36me3 levels detected by H3K36me3 CUT&Tag around gene body regions in WT and *Setd2* KO mESCs. The H3K36me3 antibody used was from Active Motif. 5 Kb windows spanning the TSS to TES of all genes determined by NCBI RefSeq were plotted. Genes were arranged by their enrichments of H3K36me3 in WT cells. Norm. RRPM, normalized reference-adjusted reads per million.

(**d**) IGV tracks presenting the enrichment of H3K36me3 by H3K36me3 CUT&Tag in WT and *Setd2* KO mESCs using the antibody from Active Motif. Three different chromatin loci were shown. Red boxes indicated the promoter regions. Blue boxes indicated gene body regions.

(**e**) The normalized read distribution profiles of H3K36me3 CUT&Tag spanning 5 Kb of gene bodies in WT and *Setd2* KO mESCs. The average read density at all genes determined by NCBI RefSeq was plotted. IgG was used as the negative control for the enrichment of H3K36me3. TSS, transcription start site. TES, transcription end site. The reads were not normalized to *E.coli* DNA.

(**f**) Same as in (e), except H3K36me3 antibody from Active Motif was used.

(**g**)Heatmaps illustrating H3K36me3 levels detected by H3K36me3 N-ChIP-Rx around gene body regions in WT and *Setd2* KO mESCs.

(**h**) The normalized read distribution profiles of H3K36me3 native ChIP-seq of ENCODE data spanning 5 Kb of gene bodies in mESCs (ENCSR000CGR [https://www.encodeproject.org/experiments/ENCSR000CGR/]). The average read density at all genes determined by NCBI RefSeq was plotted. TSS, transcription start site. TES, transcription end site.

(**i**) Heatmaps illustrating H3K36me3 levels detected in ENCODE data (ENCSR000CGR [https://www.encodeproject.org/experiments/ENCSR000CGR/]) around gene body regions in WT mESCs. 5 Kb windows spanning the TSS to TES of all genes determined by NCBI RefSeq were plotted. Genes were arranged by the enrichment of H3K36me3.

(**j**) As in (i), except H3K36me3 X-ChIP-Rx was conducted.

(**k**) The normalized read distribution profiles of Input signals spanning 1 Kb of TSS in WT mESCs in N-ChIP-Rx. The average read density at all genes determined by NCBI RefSeq was plotted.
(**l**) Same as in (k), except Input signals of X-ChIP-Rx were used.
(**m**) Heatmaps showing Pol II levels detected by Pol II CUT&Tag in WT mESCs. Nuclei were treated with Flavopiridol at 1 μM, THZ1 at 1 μM or high salt (300 mM NaCl) for 30 minutes before tagmentation. 5 Kb windows spanning the TSS to TES of all genes determined by NCBI RefSeq were plotted. Genes were arranged by the enrichment of H3K36me3 in nontreated cells.
(**n**) Same as in (m), except H3K36me3 CUT&Tag signals were shown.

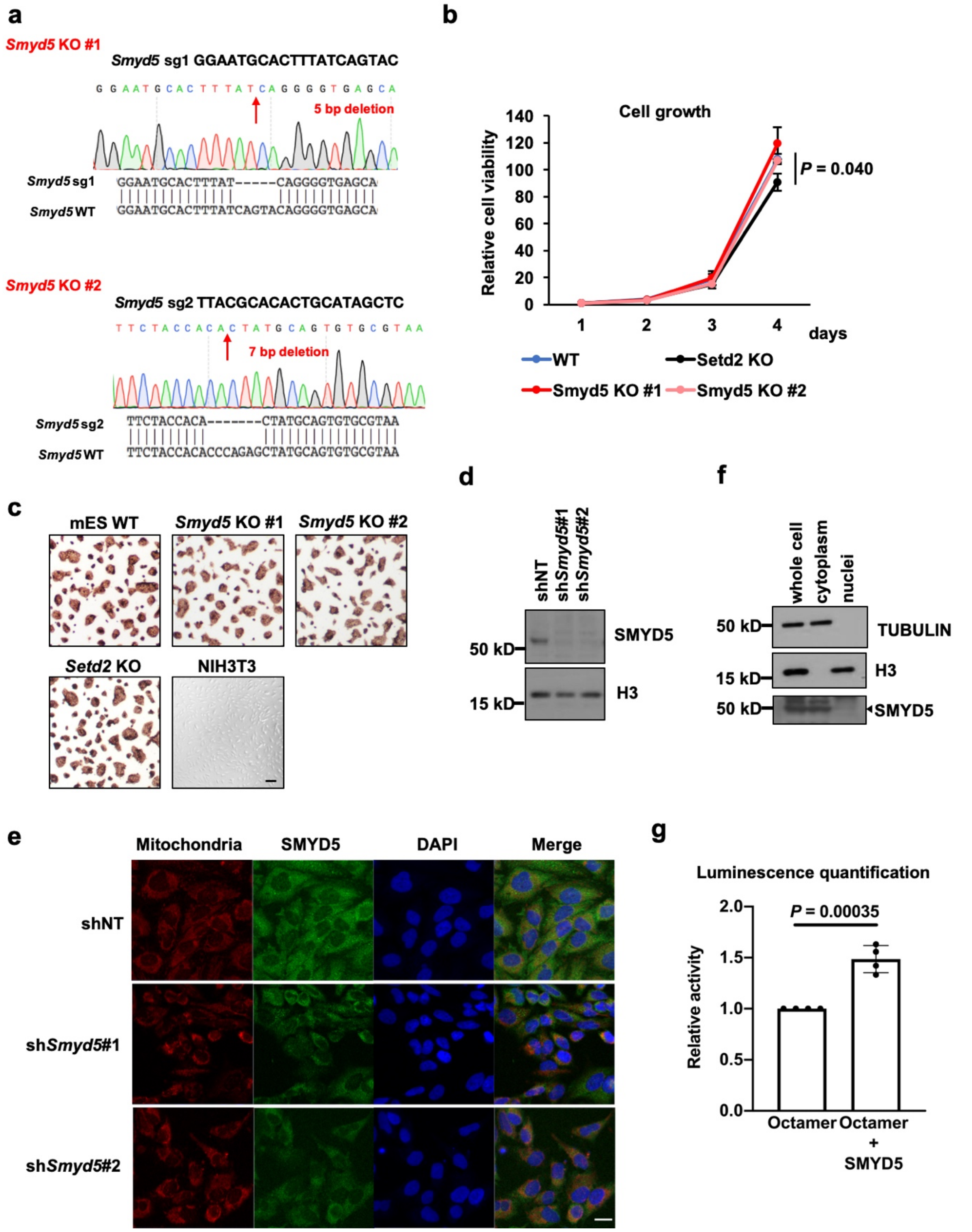

Supplementary Figure 2. *Smyd5* KO doesn't affect cell proliferation and alkaline phosphatase activity.

(**a**) Two alleles of *Smyd5* were mutated in KO clones. The Sanger sequencing results of the *Smyd5* gene locus were shown. 5 bp deletion and 7 bp deletion were identified at both alleles in *Smyd5* KO #1 and #2 clones, respectively.

(**b**) Cell proliferation was not affected in *Smyd5* KO mESCs. *Setd2* KO #2 mESCs were used as a control for the proliferation. Data represented the mean ± SD (N = 2 independent replications). *P* value was determined by Student's t-test, one-sided. Source data are provided as a Source Data file.

(**c**) Alkaline phosphatase activities were maintained in *Smyd5* KO mESCs. *Setd2* KO #2 mESCs and NIH3T3 cells were used as controls. Scale bar,100 μm. Two independent experiments were performed.

(**d**) Western blotting showing the knockdown efficiency of Smyd5 shRNAs in Hela cells. NT, non-targeting control. Two independent experiments were performed. Source data are provided as a Source Data file.

(**e**) Mitochondria and SMYD5 were co-stained in *Smyd5* knockdown Hela cells. Mitochondria was stained by MitoTracker Red, SMYD5 was stained by SMYD5 antibody. Scale bar, 20 μm.

(**f**) SMYD5 was in the nuclear fraction. Whole-cell lysate, nuclear fraction and cytoplasmic fraction were analyzed by the indicated antibodies. TUBULIN was used as the marker for cytoplasmic fraction and H3 was used as the marker for nuclear fraction. Two independent experiments were performed. Source data are provided as a Source Data file.

(**g**) End-point HMT assays of the equal amount of WT octamers with or without SMYD5. After the reaction, SAM was transferred to SAH which was detected by the MTase-Glo™ assay. Each assay was repeated at least three times with similar results. N = 4 independent experiments. Data are mean ± SD. *P* values were calculated by one-way ANOVA. Source data are provided as a Source Data file.

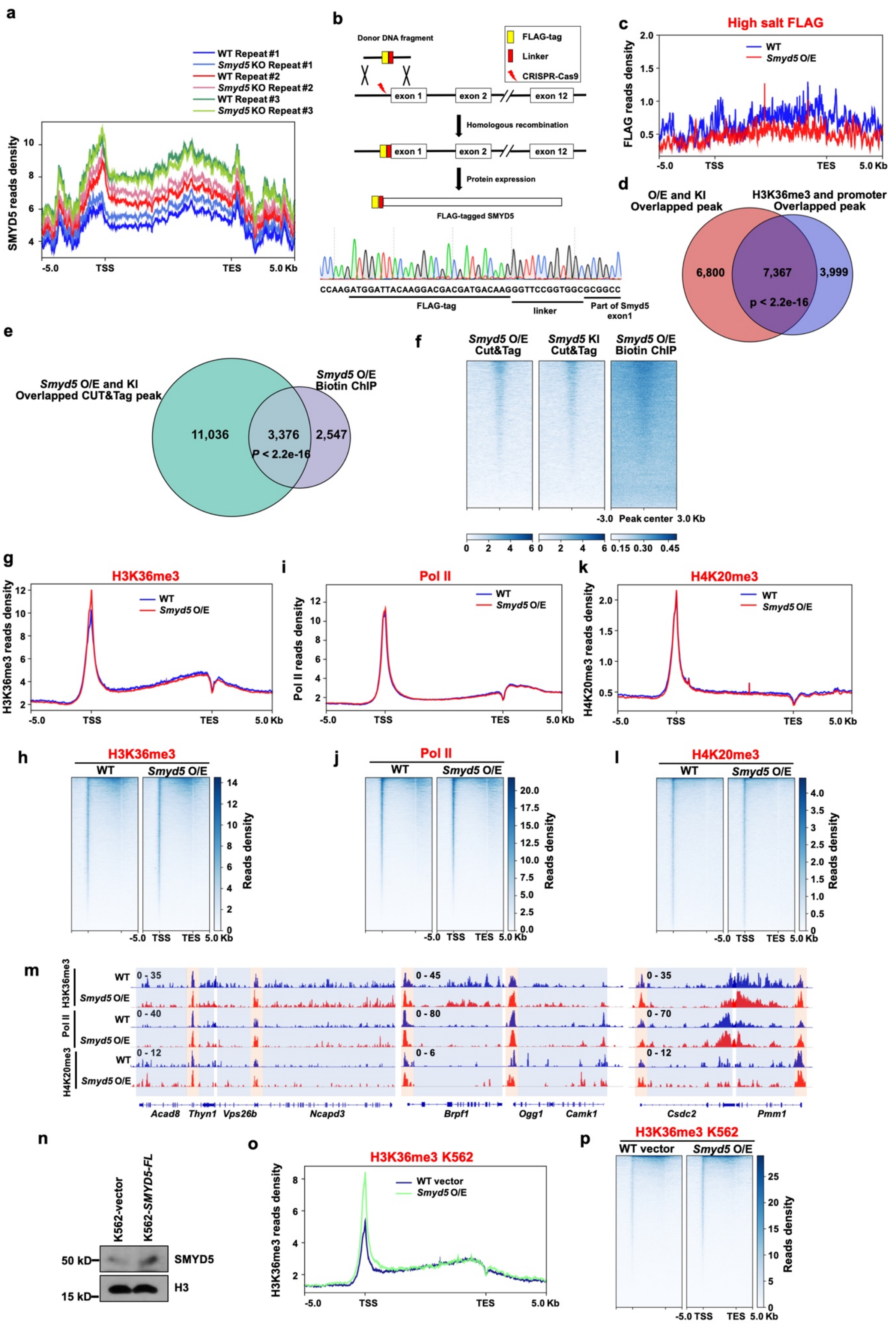

Supplementary Figure 3. SMYD5 is localized at the promoters.

(**a**) The normalized read distribution profiles of SMYD5 CUT&Tag data spanning 5 Kb of gene bodies in parental and *Smyd5* KO mESCs. SMYD5 antibody was used for the CUT&Tag. The average read density at all genes determined by NCBI RefSeq was plotted. TSS, transcription start site. TES, transcription end site. Data representing three times repeat of sequencing results.

(**b**) Schema showing the knock-in strategy. FLAG-tag was inserted to the 5' end of exon 1 of *Smyd5* by CRISPR/Cas9 directed homologous recombination. Sanger sequencing results of the knock-in mESCs were shown at the bottom indicating a homozygous clone.

(**c**) The normalized read distribution profiles of FLAG CUT&Tag data spanning 5 Kb of gene bodies in parental and *FLAG-Smyd5* over-expression mESCs. Nuclei were treated with high salt (300 mM NaCl) right before tagmentation. The average read density at all genes determined by NCBI RefSeq was plotted. TSS, transcription start site. TES, transcription end site. Two repeats for each sequencing were merged for the analysis.

(**d**) Venn diagram illustrating the overlap of SMYD5 and H3K36me3 peaks. To generate reproducible results, the overlapped peaks of FLAG-SMYD5 peaks detected in *FLAG-Smyd5* over-expression and those in FLAG tag knock-in mESCs were used. H3K36me3 peaks which were overlapped with promoters were used. *P* value was determined by Fisher's exact statistical test, two-sided. O/E, over-expression. KI, FLAG tag knock-in.

(**e**) Venn diagram showing the overlap of SMYD5 CUT&Tag and ChIP-seq peaks. *P* value was determined by Fisher's exact statistical test, two-sided.

(**f**) Heatmaps illustrating H3K36me3 levels detected by H3K36me3 CUT&Tag and ChIP-seq at overlapped peaks of SMYD5 CUT&Tag in *Smyd5* overexpression and knock-in mESCs.

(**g**) The normalized read distribution profiles of H3K36me3 CUT&Tag data spanning 5 Kb of gene bodies in parental and *FLAG-Smyd5* over-expression mESCs. The average read density at all genes determined by NCBI RefSeq was plotted. TSS, transcription start site. TES, transcription end site.

(**h**) Heatmaps illustrating H3K36me3 levels detected by H3K36me3 CUT&Tag around gene body regions in parental and *FLAG-Smyd5* over-expression mESCs. 5 Kb windows spanning the TSS to TES of all genes determined by NCBI RefSeq were plotted. Genes were arranged by their enrichments of H3K36me3 in WT cells.

(**i**) Same as in (h), except Pol II levels detected by Pol II CUT&Tag were plotted.

(**j**) Same as in (i), except Pol II levels detected by Pol II CUT&Tag were plotted.

(**k**) Same as in (h), except H4K20me3 levels detected by H4K20me3 CUT&Tag were plotted.

(**l**) Same as in (i), except H4K20me3 levels detected by H4K20me3 CUT&Tag were plotted.

(**m**) IGV tracks presenting the enrichments of H3K36me3, Pol II, and H4K20me3 by CUT&Tag in parental and *FLAG-Smyd5* over-expression mESCs. Three different chromatin loci were shown. Red boxes indicated the promoter regions. Blue boxes indicated gene body regions.

(**n**) Western blotting showing the expression of SMYD5 in K562 cells. Two independent experiments were performed. Source data are provided as a Source Data file.

(**o**) The normalized read distribution profiles of H3K36me3 CUT&Tag spanning 5 Kb of gene bodies in K562 cells transfected with vector control and *Smyd5* overexpression plasmids. The average read density at all genes determined by NCBI RefSeq was plotted.

(**p**) Heatmaps illustrating H3K36me3 levels detected by H3K36me3 CUT&Tag around gene body regions in K562 cells transfected with vector control and *Smyd5* overexpression plasmids.

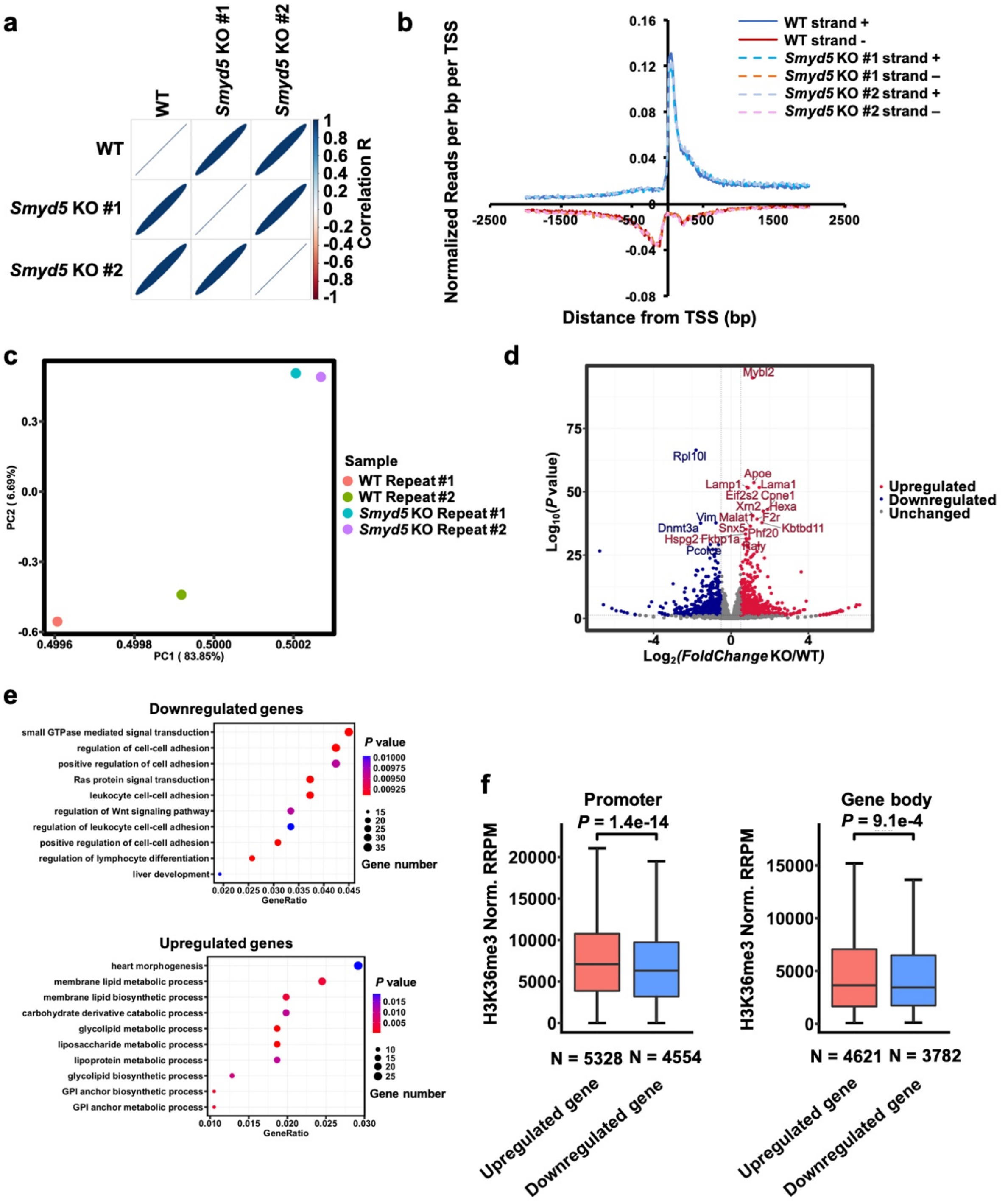

Supplementary Figure 4. Knockout of *Smyd5* changes gene expression in mESCs.

(**a**) The Pearson correlations among GRO-seq libraries constructed from WT and *Smyd5* KO mESCs.

(**b**) Comparison of GRO-seq signals among WT and *Smyd5* KO mESCs. Sense (strand +) and antisense (strand -) transcripts associated with TSS were shown.

(**c**) The principal component analysis (PCA) plot of gene expression data from WT and *Smyd5* KO #1 mESCs. Two replicates of each cell line were presented.

(**d**) Gene expression levels in WT and *Smyd5* KO mESCs. To get the reproducible results, RNA sequencing data from two replicates of each cell line were merged. Red dots, the upregulated genes defined as a $\log_2(foldchang) > 0.5$ and P value less than 0.05. Blue dots, the downregulated genes defined as a $\log_2(foldchang) < 0.5$ and P value less than 0.05.
(**e**) GO analysis result of down- and upregulated genes in *Smyd5* KO mESCs. The changed genes were defined as in (d). The top 10 GO terms ranked by P values were shown. P value was determined by Hypergeometric test, two-sided.
(**f**) Boxplots showing the enrichment of H3K36me3 at promoters and gene body regions. Genes were separated into upregulated genes or downregulated genes as defined in (d). P values were calculated by Student's t-test, two-sided. The boxes were drawn from lower quartile (Q1) to upper quartile (Q3) with the middle line denoting the median, and whiskers with maximum 1.5 IQR. Source data are provided as a Source Data file.

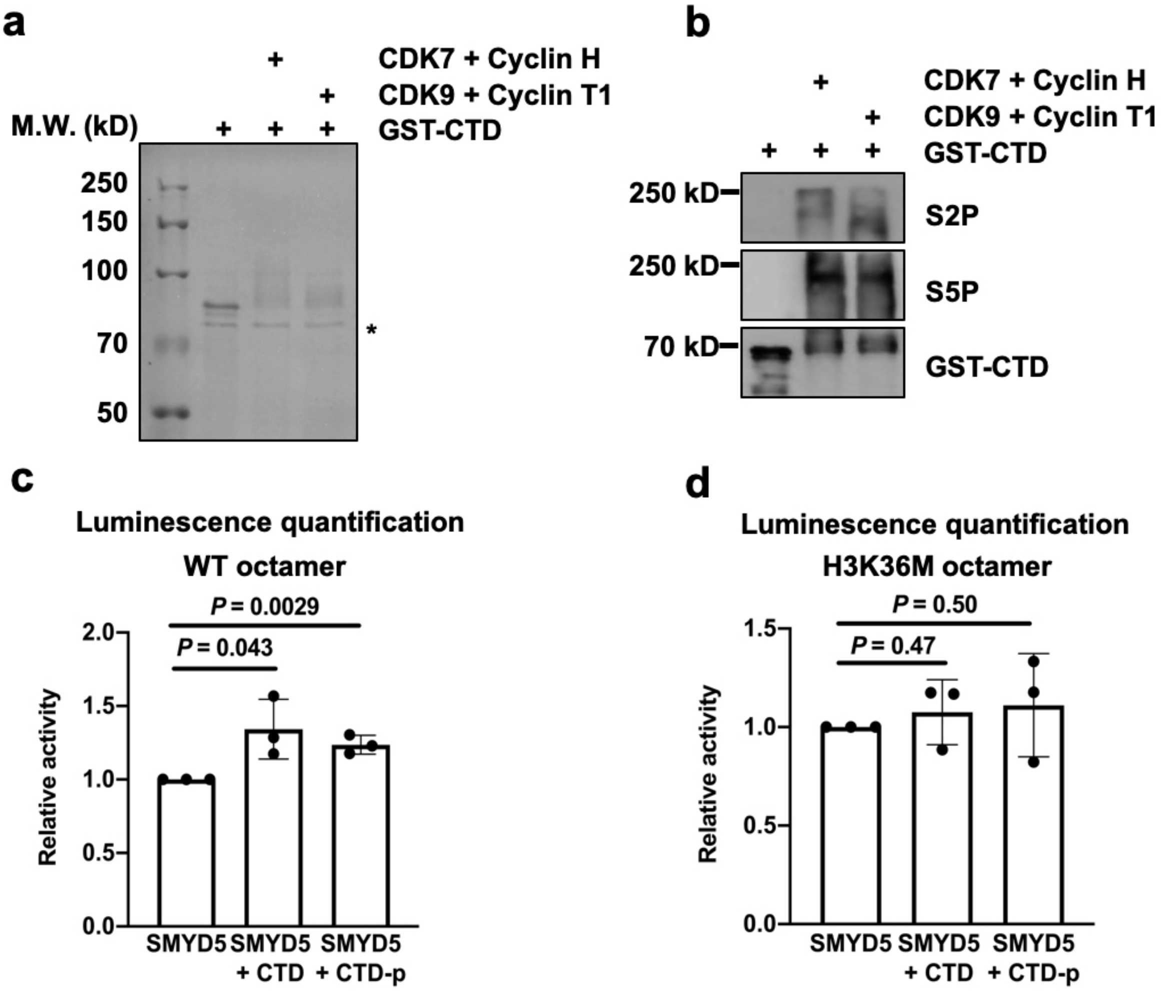

Supplementary Figure 5. Pol II CTD increases the enzymatic activity of SMYD5.

(**a**) Coomassie Brilliant Blue staining of purified Pol II CTD and phosphorylated Pol II CTD. Recombinant GST tagged Pol II CTD was purified and incubated with CDK7/Cyclin H and CDK9/Cyclin T1 complex, respectively. Proteins were analyzed by SDS-PAGE and Coomassie Brilliant Blue staining. Star indicated a non-specific protein. Two independent experiments were performed.

(**b**) Pol II CTD was phosphorylated in vitro. Samples as in (a) were analyzed by Western blotting using the indicated antibodies. Two independent experiments were performed. Source data are provided as a Source Data file.

(**c**) End-point HMT assays of SMYD5 against an equal amount of WT octamers. Phosphorylated or unphosphorylated Pol II CTD was added to analyze the changes of enzymatic activities of SMYD5. After the reaction, SAM was transferred to SAH which was detected by the MTase-Glo™ assay. Each assay was repeated at least three times with similar results. N = 3 independent experiments. Data are mean ± SD. *P* values were calculated by one-

way ANOVA. CTD, Pol II CTD. CTD-p, phosphorylated Pol II CTD. Source data are provided as a Source Data file.

(**d**) Same as in (c), except H3K36M octamers were used as substrates. Source data are provided as a Source Data file.

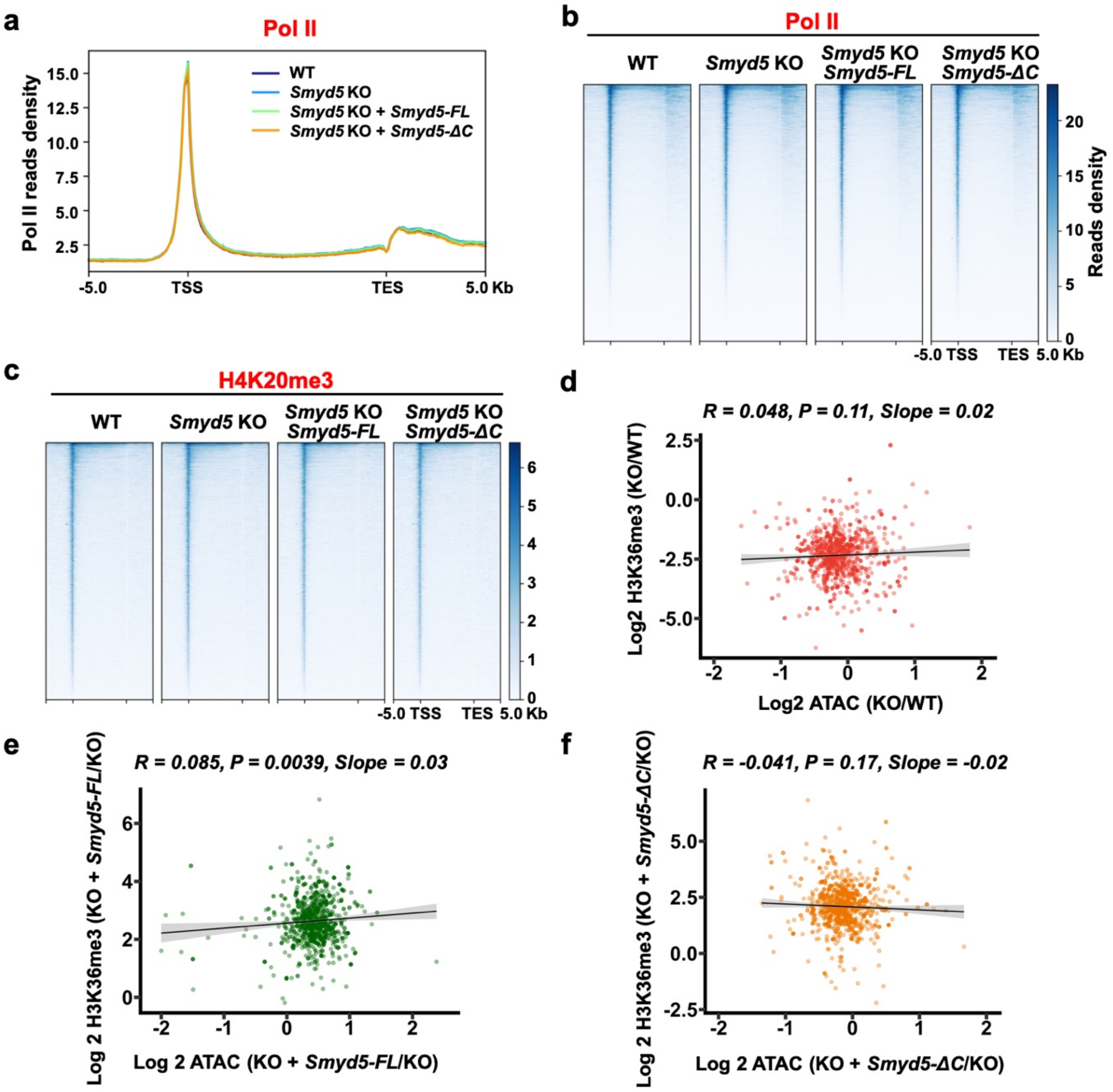

Supplementary Figure 6. Pol II and H4K20me3 at promoters are not altered when *Smyd5* is knocked out.

(**a**) The normalized read distribution profiles of Pol II CUT&Tag signals. The average read density at all genes determined by NCBI RefSeq was plotted. TSS, transcription start site. TES, transcription end site.

(**b**) Heatmaps illustrating Pol II levels around gene body regions. 5 Kb windows spanning the TSS to TES of all genes determined by NCBI RefSeq were plotted. Genes were arranged by their enrichments of H3K36me3 in WT cells.

(**c**) Heatmaps illustrating H4K20me3 levels around gene body regions.

(**d**) The difference in read densities of H3K36me3 at promoters between *Smyd5* KO and WT mESCs relative to that of ATAC-seq. R, correlation coefficients that were assessed by Pearson

product moment correlation. *P* values were calculated by paired t-test, two-sided. Confidence interval shows the SEM. Source data are provided as a Source Data file.

(**e**) Same as in (d), except signals between *Smyd5-FL* reconstituted and KO cells were analyzed. Source data are provided as a Source Data file.

(**f**) Same as in (d), except signals between *Smyd5-ΔC* reconstituted and KO cells were analyzed. Source data are provided as a Source Data file.

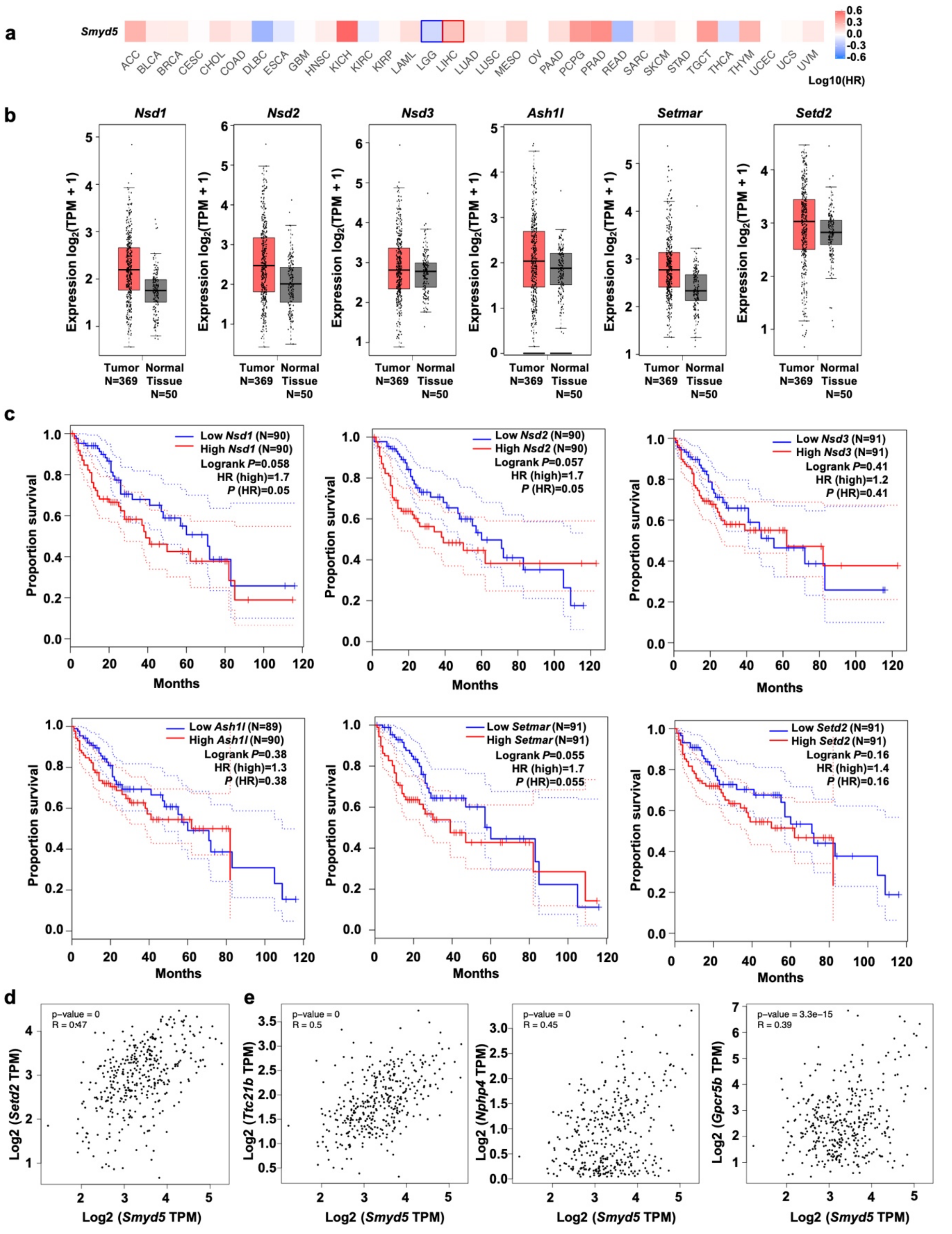

Supplementary Figure 7. Expression and correlation with survival of H3K36 methyltransferases in liver hepatocellular carcinoma.

(**a**) *Smyd5* is correlated with survival rates in LGG and LIHC. The significantly associated tumors were circled. Summary of the Kaplan-Meier analysis of overall survival in tumor cases based on *Smyd5* level. Patient data were from the TCGA database through GEPIA analysis. HR, hazard rate ratio. ACC, Adrenocortical carcinoma. BLCA, Bladder Urothelial Carcinoma.

BRCA, Breast invasive carcinoma. CESC, Cervical squamous cell carcinoma and endocervical adenocarcinoma. CHOL, Cholangio carcinoma. COAD, Colon adenocarcinoma. DLBC, Lymphoid Neoplasm Diffuse Large B-cell Lymphoma. ESCA, Esophageal carcinoma. GBM, Glioblastoma multiforme. HNSC, Head and Neck squamous cell carcinoma. KICH, Kidney Chromophobe. KIRC, Kidney renal clear cell carcinoma. KIRP, Kidney renal papillary cell carcinoma. LAML, Acute Myeloid Leukemia. LGG, Brain Lower Grade Glioma. LIHC, Liver hepatocellular carcinoma. LUAD, Lung adenocarcinoma. LUSC, Lung squamous cell carcinoma. MESO, Mesothelioma. OV, Ovarian serous cystadenocarcinoma. PAAD, Pancreatic adenocarcinoma. PCPG, Pheochromocytoma and Paraganglioma. PRAD, Prostate adenocarcinoma. READ, Rectum adenocarcinoma. SARC, Sarcoma. SKCM, Skin Cutaneous Melanoma. STAD, Stomach adenocarcinoma. TGCT, Testicular Germ Cell Tumors. THCA, Thyroid carcinoma. THYM, Thymoma. UCEC, Uterine Corpus Endometrial Carcinoma. UCS, Uterine Carcinosarcoma. UVM, Uveal Melanoma.

(**b**) H3K36 methyltransferases are slightly elevated without significances in LIHC tumors than normal tissues. The expression levels were generated from the TCGA database through GEPIA analysis. TPM, transcript per million. The boxes were drawn from lower quartile (Q1) to upper quartile (Q3) with the middle line denoting the median, and whiskers with maximum 1.5 IQR.

(**c**) Kaplan-Meier analysis of overall survival in LIHC cases. Patient data were from the TCGA database through GEPIA analysis. HR, hazard rate ratio.

(**d**) The Pearson correlation between *Smyd5* and *Setd2* in LIHC tumors. The expression levels were generated from the TCGA database through GEPIA analysis. TPM, transcript per million. *P* values were calculated by paired t-test, two-sided.

(**e**) The Pearson correlation of *Smyd5* with *Ttc21b*, *Nphp4* and *Gpcr5b* in LIHC tumors. The expression levels were generated from the TCGA database through GEPIA analysis. TPM, transcript per million. *P* values were calculated by paired t-test, two-sided.

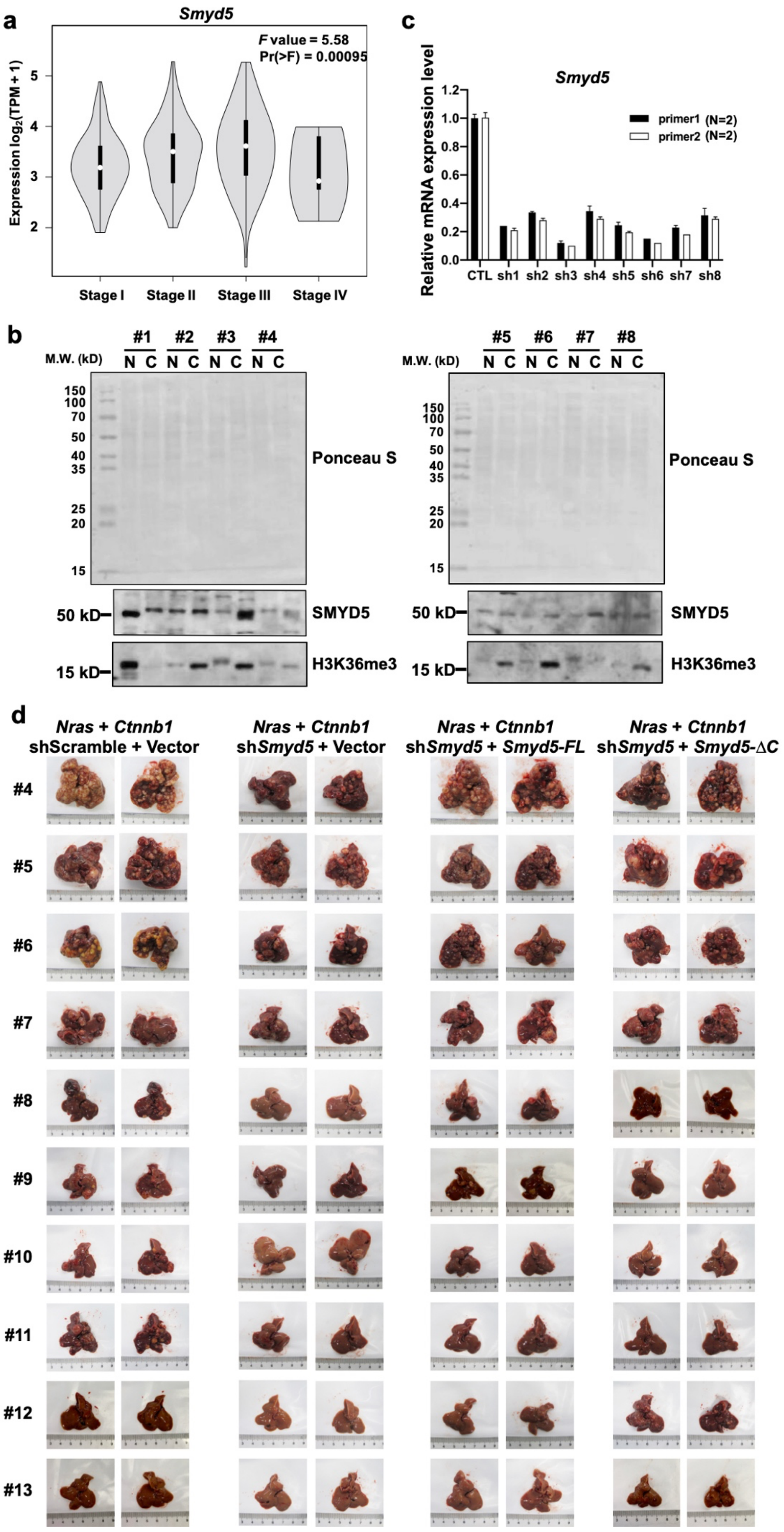
a
Smyd5
F value = 5.58
Pr(>F) = 0.00095
Expression log2(TPM + 1)
5
4
3
2
Stage I
Stage II
Stage III
Stage IV
c
Smyd5
Relative mRNA expression level
1.2
1.0
0.8
0.6
0.4
0.2
0.0
primer1 (N=2)
primer2 (N=2)
CTL
sh1
sh2
sh3
sh4
sh5
sh6
sh7
sh8
b
M.W. (kD)
#1
#2
#3
#4
#5
#6
#7
#8
N C N C N C N C
150
100
70
50
40
35
25
20
15
Ponceau S
50 kD
SMYD5
15 kD
H3K36me3
d
Nras + Ctnnb1
shScramble + Vector
Nras + Ctnnb1
shSmyd5 + Vector
Nras + Ctnnb1
shSmyd5 + Smyd5-FL
Nras + Ctnnb1
shSmyd5 + Smyd5-ΔC
#4
#5
#6
#7
#8
#9
#10
#11
#12
#13

Supplementary Figure 8. *Smyd5* promotes liver tumor formation.

(**a**) *Smyd5* expression violin plots based on patient pathological stage. The expression levels of Smyd5 were generated from the TCGA database through GEPIA analysis. TPM, transcript per million. Pr(>F), *P* value for F statistics. *P* values were calculated by one-way ANOVA. The violin plots were drawn from lower quartile (Q1) to upper quartile (Q3) with the middle line denoting the median, and whiskers with maximum 1.5 IQR.

(**b**) Western blotting showing the levels of SMYD5 and H3K36me2 in non-cancer and cancer tissues of liver cancer patients. The loading of each samples was stained with Ponceau S. The levels of SMYD5 and H3K36me3 were detected by the antibody. N, non-cancer tissue; C, cancer tissue. Samples from 8 different patients were analyzed. Two independent experiments were performed. Source data are provided as a Source Data file.

(**c**) The expression levels of *Smyd5*were analyzed via RT-qPCR. The expression level of *Smyd5* in scramble shRNA treated control cells was set as 1. The data are represented by the mean ± SD. N=2 independent experiments. CTL, Scramble shRNA control. Source data are provided as a Source Data file.

(**d**) *Smyd5* participated in the tumorigenesis of liver tumors. Mice were sacrificed 90-100 days after injection and livers were pictured. Ten representative livers in each cohort were shown.

Supplementary Table 1. Correlations between two replicates of epigenomic sequencing results. A 1 kb sliding window across the whole genome was used to calculate the Pearson product moment correlation for epigenomic sequencing.

Samples	Correlation
H3K36me3_WT	0.79
H3K36me3_Setd2_KO	0.72
IgG_WT	0.78
H3K36me3_WT_nativeChIP	0.82
H3K36me3_Setd2 KO_native_ChIP	0.81
H3K36me3_WT_FixChIP	0.77
H3K36me3_Setd2 KO_FixChip	0.78
Pol2	0.9
Pol2_Flavopiridol	0.9
Pol2_THZ1	0.9
Pol2_High_Salt	0.77
H3K36me3_Flavopiridol	0.8
H3K36me3_THZ1	0.8
H3K36me3_High_Salt	0.96
H3K36me3_WT_AM	0.69
H3K36me3_Setd2_KO_AM	0.42
H3K36me3_Smyd5_KO1	0.81
H3K36me3_Smyd5_KO2	0.84
FLAG_WT	0.76
FLAG_Smyd5_OE	0.79
FLAG_Smyd5_KI	0.69
FLAG_WT_High_Salt.bw	0.71
FLAG_Smyd5_OE_High_Salt	0.78
H3K36me3_WT_forSmyd5_OE	0.86
H3K36me3_Smyd5_OE	0.84
Pol2_WT	0.87
Pol2_Smyd5_OE	0.91
H4K20me3_WT	0.87
H4K20me3_Smyd5_OE	0.86
FLAG_WT_rescue	0.76
FLAG_Smyd5_KO	0.79
FLAG_Smyd5_KO_Smyd5_FL	0.77
FLAG_Smyd5_KO_Smyd5_delC	0.75
H3K36me3_WT_rescue	0.86
H3K36me3_Smyd5_KO_rescue	0.85
H3K36me3_Smyd5_KO_Smyd5_FL	0.84
H3K36me3_Smyd5_KO_Smyd5_delC	0.8
Pol2_WT_rescue	0.87

Samples	Correlation
Pol2_Smyd5_KO	0.9
Pol2_Smyd5_KO_Smyd5_FL	0.91
Pol2_Smyd5_KO_Smyd5_delC	0.88
H4K20me3_WT_rescue	0.87
H4K20me3_Smyd5_KO	0.88
H4K20me3_Smyd5_KO_Smyd5_FL	0.88
H4K20me3_Smyd5_KO_Smyd5_delC	0.84
H3K9me3	0.79
ATAC_WT	0.67
ATAC_Smyd5 KO	0.65
ATAC_ Smyd5_KO_Smyd5_FL	0.74
ATAC_ Smyd5_KO_Smyd5_delC	0.80
H3K36me3_ K562	0.71
H3K36me3_ K562_Smyd5OE	0.64

Supplementary Table 2. Raw data for first screening.

**Gene**	**log_2(Promoters+1) (KO/WT)**	**log_2(Genebody+1) (KO/WT)**	**Index**	**Threshold**
Ash1l	0.925	1.014	0.912	Decreased
Kmt2c	1.150	1.102	1.044	Unchanged
Prdm9 #1	1.108	1.049	1.056	Unchanged
Setd3	1.039	0.961	1.081	Unchanged
Setd7	1.020	0.997	1.023	Unchanged
Setmar #1	0.992	0.977	1.016	Unchanged
Smyd1	0.893	0.835	1.069	Unchanged
Smyd2	1.058	1.068	0.990	Unchanged
Smyd5 #1	0.823	1.152	0.714	Decreased
Zmynd1 #1	0.936	1.110	0.843	Decreased
Zmynd10	1.017	1.156	0.880	Decreased
Zmynd11 #1	0.979	1.121	0.873	Decreased
Zmynd16	1.018	1.082	0.941	Unchanged
Zmynd19 #1	0.989	1.137	0.869	Decreased
Zmynd8	1.095	1.119	0.979	Unchanged
Zmynd20	1.471	0.808	1.821	Increased
Kmt2d #1	0.920	1.018	0.903	Decreased
Phf8	0.930	0.991	0.938	Unchanged
Prdm11	0.908	0.755	1.203	Increased
Prdm13	0.891	1.031	0.865	Decreased
Prdm15	1.067	0.988	1.080	Unchanged
Zmynd13	0.835	1.071	0.780	Decreased
Kmt2d #2	1.038	0.888	1.169	Increased

Gene	log2(Promoters+1) (KO/WT)	log2(Genebody+1) (KO/WT)	Index	Threshold
G9a	0.856	1.085	0.789	Decreased
Prdm14	0.778	1.037	0.751	Decreased
Setd1a #1	0.895	1.070	0.836	Decreased
Setd1a #2	0.803	0.978	0.821	Decreased
Setd6	0.839	1.054	0.796	Decreased
Brd4	0.964	0.961	1.003	Unchanged
Kmt2e	0.913	0.938	0.974	Unchanged
Setd8 #1	0.894	0.955	0.937	Unchanged
Setd8 #2	0.965	0.924	1.044	Unchanged
Kmt2d #3	1.085	1.061	1.023	Unchanged
Prdm13	0.982	0.918	1.070	Unchanged
Prdm9 #2	1.007	1.004	1.004	Unchanged
Setmar #2	0.958	0.999	0.959	Unchanged
Smyd5 #2	0.899	1.064	0.845	Decreased
Zmynd11 #2	1.050	1.025	1.024	Unchanged
Zmynd17	1.060	1.079	0.983	Unchanged
Zmynd19 #2	1.103	1.029	1.072	Unchanged
Setd2 #2	0.840	0.710	1.184	Increased
Zmynd1 #2	0.725	1.027	0.706	Decreased
Setd2 #1	1.022	0.771	1.326	Increased
Setdb2	0.885	0.899	0.984	Unchanged
Zmynd12	1.016	0.993	1.023	Unchanged
Setd1b	0.788	0.940	0.838	Decreased
Mmset	1.098	1.023	1.073	Unchanged
Nsd3	0.922	0.721	1.279	Increased
Top1	1.024	0.958	1.068	Unchanged

Supplementary Table 3. Raw data for second screening.

Gene	log2(Promoters+1) (KO/WT)	log2(Genebody+1) (KO/WT)	Index	Threshold	Repeat
Kmt2a	1.045	1.045	0.999	Unchange	Different
Setd4	1.317	1.147	1.148	Increased	Different
Zmynd9	1.043	1.115	0.936	Unchange	Different
Prdm1 #1	0.816	1.095	0.745	Decreased	Different
Setd5	1.071	1.158	0.925	Decreased	Different
Suv420h1 #1	0.978	1.043	0.937	Unchange	Different
Zmynd17	0.986	1.066	0.925	Unchange	Different
Nsd3 #1	1.732	0.933	1.857	Increased	Increase
Zmynd1	1.696	0.814	2.083	Increased	Different
Zmynd21	1.526	1.025	1.488	Increased	Different

Gene	log$_2$(Promoters+1) (KO/WT)	log$_2$(Genebody+1) (KO/WT)	Index	Threshold	Repeat
Prdm12	0.862	0.824	1.046	Unchange	Different
Prdm16	0.994	0.885	1.123	Increased	Different
Prdm3	1.039	0.966	1.076	Unchange	Different
Prdm4	1.232	1.000	1.232	Increased	Different
Prdm5	1.271	0.941	1.351	Increased	Different
Prdm6	1.215	0.882	1.377	Increased	Different
Setd1b	0.867	0.942	0.920	Unchange	Different
Suv420h2	1.120	0.999	1.121	Increased	Different
Zmynd15	1.024	0.982	1.042	Unchange	Different
Zmynd6	1.029	0.777	1.324	Increased	Different
Prdm2	0.974	0.906	1.075	Unchange	Different
Phf2	0.688	0.946	0.728	Decreased	Different
Prdm10	0.830	1.120	0.741	Decreased	Different
Prdm8	0.793	1.014	0.782	Decreased	Different
Zmynd12 #1	0.926	1.447	0.640	Decreased	Different
Zmynd2	0.596	0.960	0.621	Decreased	Different
Prdm1 #2	1.009	1.078	0.936	Unchange	Different
Set1b	0.961	1.054	0.912	Decreased	Different
Zmynd13	1.209	0.984	1.229	Increased	Different
Zmynd12 #2	0.970	0.931	1.043	Unchange	Different
Kmd4d	0.905	0.870	1.040	Unchange	Different
Nsd3 #2	0.920	0.925	0.995	Unchange	Different
Setd2 #2	0.970	0.803	1.209	Increased	Increase
Smyd5	0.816	0.973	0.839	Decreased	Decrease
Zmynd1	1.134	0.998	1.137	Unchange	Different
Suv420h1 #2	1.055	1.010	1.044	Unchange	Different
Mmset	1.107	1.025	1.080	Unchange	Different
Setd2 #1	1.123	0.794	1.414	Increased	Increase

Supplementary Table 4. SMYD5 regulated genes in mESC.

Gene	Gene	Gene	Gene	Gene	Gene	Gene
1700030C1	Camkk2	Fam169a	Gpcpd1	Lpcat3	Ppfibp1	Taco1
4933404O1	Ckb	Fbxo4	Gprc5b	Mettl7a1	Ppp3ca	Tbc1d10c
Acsf2	Col20a1	Fcgr2b	Gpsm3	Mgll	Ptpn21	Thap7
Adgrb2	Crabp1	Fgr	Hck	Mmp14	Rab6b	Tll1
Adgrl1	Crmp1	Fhl3	Hes6	Nab1	Rel	Tmem229b
Adrb3	Cyp2s1	Flvcr2	Hmga2	Nckap5l	Rell1	Trmt44
Arfgap3	Cyp4f16	Fn3krp	Ift81	Neat1	Rgs3	Ttc21b
Arl4a	Dapp1	Foxp1	Ihh	Nphp4	Rnd2	Tubb3
Atf7	Dixdc1	Glt1d1	Insr	Nwd2	Scara5	Ubr4

Gene	Gene	Gene	Gene	Gene	Gene	Gene
B9d1	*Dmtn*	*Gm2a*	*Kcnj12*	*Pcdhgb8*	*Slc4a8*	*Ucma*
Bbs10	*Dusp4*	*Gm8801*	*Klk10*	*Phldb2*	*Snhg11*	*Ydjc*
Cacng7	*Faap24*	*Gne*	*Lama5*	*Pknox2*	*Spn*	*Zic2*

Supplementary Table 5. Oligonucleotides used in the study.

Name	5'- 3'	Application
*Setd2*_m_sgRNA1	AGTTCCTCTCGATGTCCAAA	Knock-out
*Setd2*_m_sgRNA2	CTGGGGCTTAAGGGCTGCTA	Knock-out
*Smyd5*_m_sgRNA1	GGAATGCACTTTATCAGTAC	Knock-out
*Smyd5*_m_sgRNA2	TTACGCACACTGCATAGCTC	Knock-out
*Sdha*_m_RT_F	ATATGGTGCAGAAGCTCGGAA	Gene expression
Sdha _m_RT_R	GTTCCCCAAACGGCTTCTTC	Gene expression
*Smyd5*_m_RT_F	AAGTCCGTTATGTGGACAGCATC	Gene expression
*Smyd5*_m_RT_R	AGTCAGCCTCTGGGCATTCT	Gene expression
sgRNA-FLAG-*Smyd5* KI	CACATGGAGGCCGCCATCT	sgRNA for knock-in
FLAG-*Smyd5* KI oligo	GGCGGGGCCTCCCGGGACGGGGTCAAGG GTCAGAAGGCAGAGGCGTGCCCAAGATG GATTACAAGGACGACGATGACAAGGGTTC CGGTGGCGCGGCCTCCATGTGCGACGTGT TCTCCTTCTGCGTGGGCGTGGCGGACCG	ssDNA for knock-in FLAG tag
*Smyd5*_m_sh1_F	CCGGGTTCCGTGGAAGTCCGTTATGCTCG AGCATAACGGACTTCCACGGAACTTTTTC	Knock-down
*Smyd5*_m_sh1_R	AATTGAAAAAGTTCCGTGGAAGTCCGTTA TGCTCGAGCATAACGGACTTCCACGGAAC	Knock-down
*Smyd5*_m_sh2_F	CCGGCTCCTATTGACTGGCTATTAACTCGA GTTAATAGCCAGTCAATAGGAGTTTTTC	Knock-down
*Smyd5*_m_sh2_R	AATTGAAAAACTCCTATTGACTGGCTATT AACTCGAGTTAATAGCCAGTCAATAGGAG	Knock-down
*Smyd5*_m_sh3_F	CCGGTACTGCCCTGGAGGATATTAACTCG AGTTAATATCCTCCAGGGCAGTATTTTTC	Knock-down
*Smyd5*_m_sh3_R	AATTGAAAAATACTGCCCTGGAGGATATT AACTCGAGTTAATATCCTCCAGGGCAGTA	Knock-down
*Smyd5*_m_sh4_F	CCGGGCAGGCAATTGTCCACAAACTCTCG AGAGTTTGTGGACAATTGCCTGCTTTTTC	Knock-down

Name	5'- 3'	Application
*Smyd5*_m_sh4_R	AATTGAAAAAGCAGGCAATTGTCCACAAA CTCTCGAGAGTTTGTGGACAATTGCCTGC	Knock-down
*Smyd5*_m_sh5_F	CCGGGCTCTTCAAAGAGGCCCTTTACTCG AGTAAAGGGCCTCTTTGAAGAGCTTTTTC	Knock-down
*Smyd5*_m_sh5_R	AATTGAAAAAGCTCTTCAAAGAGGCCCTT TACTCGAGTAAAGGGCCTCTTTGAAGAGC	Knock-down
*Smyd5*_m_sh6_F	CCGGGCAACCGGAGAGTTCCTTAACCTCG AGGTTAAGGAACTCTCCGGTTGCTTTTTC	Knock-down
*Smyd5*_m_sh6_R	AATTGAAAAAGCAACCGGAGAGTTCCTTA ACCTCGAGGTTAAGGAACTCTCCGGTTGC	Knock-down
*Smyd5*_m_sh7_F	CCGGTCCTGAGACTGCGAGTATAATCTCG AGATTATACTCGCAGTCTCAGGATTTTTC	Knock-down
*Smyd5*_m_sh7_R	AATTGAAAAATCCTGAGACTGCGAGTATA ATCTCGAGATTATACTCGCAGTCTCAGGA	Knock-down
*Smyd5*_m_sh8_F	CCGGATGACCGACGTGTGATGTTATCTCG AGATAACATCACACGTCGGTCATTTTTTC	Knock-down
*Smyd5*_m_sh8_R	AATTGAAAAAATGACCGACGTGTGATGTT ATCTCGAGATAACATCACACGTCGGTCAT	Knock-down
